# Toxicological inhalation studies in rats to substantiate grouping of zinc oxide nanoforms

**DOI:** 10.1186/s12989-024-00572-y

**Published:** 2024-05-17

**Authors:** Tizia Thoma, Lan Ma-Hock, Steffen Schneider, Naveed Honarvar, Silke Treumann, Sibylle Groeters, Volker Strauss, Heike Marxfeld, Dorothee Funk-Weyer, Svenja Seiffert, Wendel Wohlleben, Martina Dammann, Karin Wiench, Noömi Lombaert, Christine Spirlet, Marie Vasquez, Nicole Dewhurst, Robert Landsiedel

**Affiliations:** 1 BASF Services Europe GmbH, Berlin, Germany; 2 BASF SE, Experimental Toxicology and Ecology, Ludwigshafen am Rhein, Germany; 3 BASF SE, Analytical and Material Science, Ludwigshafen am Rhein, Germany; 4 BASF SE, Product Stewardship, Regulatory Toxicology Chemicals, Ludwigshafen am Rhein, Germany; 5 International Zinc Association, Brussels, Belgium; 6 Helix3 Inc., Morrisville, NC USA; 7 Pharmacy, Pharmacology and Toxicology, Free University of Berlin, Berlin, Germany

**Keywords:** Nanoparticles, Zinc oxide, Metal oxide, Genotoxicity, Reproductive toxicity, Developmental toxicity, Inhalation toxicity

## Abstract

**Background:**

Significant variations exist in the forms of ZnO, making it impossible to test all forms in in vivo inhalation studies. Hence, grouping and read-across is a common approach under REACH to evaluate the toxicological profile of familiar substances. The objective of this paper is to investigate the potential role of dissolution, size, or coating in grouping ZnO (nano)forms for the purpose of hazard assessment. We performed a 90-day inhalation study (OECD test guideline no. (TG) 413) in rats combined with a reproduction/developmental (neuro)toxicity screening test (TG 421/424/426) with coated and uncoated ZnO nanoforms in comparison with microscale ZnO particles and soluble zinc sulfate. In addition, genotoxicity in the nasal cavity, lungs, liver, and bone marrow was examined via comet assay (TG 489) after 14-day inhalation exposure.

**Results:**

ZnO nanoparticles caused local toxicity in the respiratory tract. Systemic effects that were not related to the local irritation were not observed. There was no indication of impaired fertility, developmental toxicity, or developmental neurotoxicity. No indication for genotoxicity of any of the test substances was observed. Local effects were similar across the different ZnO test substances and were reversible after the end of the exposure.

**Conclusion:**

With exception of local toxicity, this study could not confirm the occasional findings in some of the previous studies regarding the above-mentioned toxicological endpoints. The two representative ZnO nanoforms and the microscale particles showed similar local effects. The ZnO nanoforms most likely exhibit their effects by zinc ions as no particles could be detected after the end of the exposure, and exposure to rapidly soluble zinc sulfate had similar effects. Obviously, material differences between the ZnO particles do not substantially alter their toxicokinetics and toxicodynamics. The grouping of ZnO nanoforms into a set of similar nanoforms is justified by these observations.

**Supplementary Information:**

The online version contains supplementary material available at 10.1186/s12989-024-00572-y.

## Background

Zinc oxide (ZnO) is an inorganic compound, that is present in the earth crust as a mineral zincite, however most ZnO used commercially is produced synthetically [1]. Zinc (Zn) is one of the most abundant microelements in the human body, where it is involved in numerous signaling pathways as well as enzyme activities.

Due to its UV light absorbing, catalytical, optical, and antimicrobial properties among many others, ZnO is used in a variety of applications in cosmetics, pharmaceuticals, and other industrial applications [2–4]. In Europe, ZnO is manufactured and imported by 100,000–1,000,000 tons per year, either as nanoforms or non-nanoforms. The amount of annually produced nanoforms is estimated to be in the range of 1000–10,000 tons [5].

ZnO in nanoforms is used as a component or an additive for various purposes including the manufacture of electronic components, production of clear coatings, polymer-matrices, plastics, thermoplastics and related preparations, and cosmetic emollients used for sunscreen, skin care and pharmaceuticals preparations, and is utilized by professionals and consumers alike [3, 4]. Based on the widespread use of products containing ZnO nanoforms humans are exposed especially via inhalation or dermal contact. Dermal uptake of ZnO particles from e.g., sunscreen products is very limited [6–8]. Whereas inhalation of ZnO (for example welding fumes) is known to induce zinc fever. Besides fever, typical symptoms include throat irritation, cough, minor respiratory symptoms, metallic taste, as well as flu-like symptoms [9, 10]. Monsé and colleagues have studied the effect of ZnO inhalation in humans, showing a dose-dependent induction of acute phase response after exposure to ZnO nanoparticles as well as a more pronounced systemic inflammatory response after exposure to micron-sized ZnO than after exposure to nano-sized ZnO [10–13].

In the recent two decades, numerous in vivo and in vitro studies have examined potential health effects of ZnO forms. Table 1 gives a compilation of selected studies with ZnO (nano)forms that were carefully evaluated, with preference given to studies performed according to OECD guidelines, with well-characterized particles, and detailed reporting of methods and results. The use of “(nano)” in parentheses in the term “(nano)forms” indicates that the presented studies encompass both nano and non-nano forms (micron-sized) of ZnO.

**Table 1:** Literature collected

Study type	**References**	**Species**	**Treatment duration**	**Substance**	**Findings**
(Repeated dose) inhalation	Fraunhofer ITEM [14]	Rat	14 Days	cZnO, uZnO (NM-111, NM-110) and µZnO (NM-113)	NOAEC: 2 mg/m^3^ based on increase of PMN, LDH and other parameters in BAL, slight inflammatory change in the lung and slight degeneration of the olfactory epithelia
	Fraunhofer ITEM [15]	Rat	90 Days	cZnO (NM-111) and µZnO (NM-113)	NOAEC: 1.5 mg/m^3^; histopathology: very slight bronchioalveolar hyperplasia and increased incidences in very slight to slight infiltration of interstitial mononuclear cells in the lung at the highest tested concentration; increased activity of lactate dehydrogenase in BAL and increased numbers of lymphocytes in BAL at the highest tested concentration LOAEC: 4.5 mg/m^3^
	Fraunhofer ITEM [16]	Rat	5 Days	cZnO (NM-111)	(Very) slight degeneration of the olfactory epithelium and alveolar accumulation of particle-laden macrophages in lungs at 8 mg/m^3^
	Adamcakova-Dodd et al. [17]	Mouse	14 Days and 13 weeks	uZnO (26 nm)	Increased Zn levels in the heart
	BASF SE [18]	Rat	5 Days + 3-week recovery	cZnO and µZnO	LOAEC: 0.5 mg/m^3^ based on increased levels of PMN, LDH, protein and other parameters in BAL, histology: minimal multifocal necrosis of the olfactory epithelium
	BASF SE [19]	Rat	28 Days (20 exposures)	µZnO	NOAEC: 0.5 mg/m^3^ based on increased PMN, LDH, protein and other parameters in BAL, increased lung weights, degeneration/regeneration of the olfactory epithelia, histiocytosis in the lungs associated with few inflammatory cells
(Repeated dose) oral	Kim et al. [20]	Rat (SD)	90 Days + 14-day recovery	Nano ZnO (positive and negative charged)	NOAEL: 31.25 mg/kg b.w./day (male/female), mean cell volume and hemoglobin, hemoglobin concentration was decreased, total protein and albumin in serum were increased; organ weights and organ / body weight ratios changed, histopathological changes in stomach, eye, and pancreas
	Park et al. [21, 22]	Rat (SD)	90 Days (OECD 408, but not explicitly stated) + 14-day recovery	Nano ZnO (30 nm positive and negative charged)	LOAEL: 125 mg/kg b.w./day (male/female), decreased hemoglobin, hematocrit, mean corpuscular volume, mean corpuscular hemoglobin concentration; histopathology: retinal atrophy of eyes, non-neoplastic inflammation and epithelial degeneration/regeneration in stomach, cell apoptosis and inflammation in pancreas at 500 mg/kg b.w.; recovery was observed after 2 weeks. Tmax was 4–6 h, t1/2 in blood was 1–13 h
(Repeated dose) dermal	Ryu et al. [23]	Rat (SD)	90 Days (OECD 411), 14-day recovery	Nano ZnO (20 nm negative charged)	NOAEL: 1000 mg/kg b.w.; no systemic toxicity, dose-dependent inflammation of the skin at the application site
	Surekha et al. [24]	Rat (SD)	28 Days (OECD 410), 6 h/day, 5 days/week	ZnO	NOAEL: 2000 mg/kg b.w
Developmental toxicity	BASF SE [25]	Rat (WI)	GD 6 to 19, OECD 414 (inhalation)	cZnO (NM-111)	NOAEC 7.5 mg/m^3^; daily inhalation exposure to 0.3, 1.5 and 7.5 mg/m^3^. No developmental toxicity; toxicity only relates to maternal local toxicity
	Schlicker and Cox [26]	Rat	Max. 21 days before mating till fetal age of 15 and 16 days (diet)	ZnO	NOAEL: 0.2% in diet. Growth reduction, variable degrees of death and resorption at 0.4% in diet
	Ketcheson et al. [27]	Rat (SD)	GD 1 to PND 14 (diet)	ZnO	Increased zinc content and reduced iron and copper content at 0.5% ZnO in diet. No developmental toxicity
	Hong et al. [28, 29]	Rat (SD)	GD 5 to 19 (daily gavage), OECD 414	nano ZnO	NOAEL (maternal): 100 mg/kg b.w., NOAEL (fetal): 400 mg/m^3^ or 200 mg/m^3^ depending on vehicle, no fetal developmental toxicity was observed
Genotoxicity	Li et al. [30]	TA1535, TA1537, TA98, TA100, TA102	Ames test, with or without metabolic activation	Nano ZnO no surface treatment	No cytotoxicity, negative
	Landsiedel et al. [31]	See above	Ames test	Nano ZnO (Z-cote max)	Up to 5000 µg/plate, no cytotoxicity, negative
	Fraunhofer ITEM [32]	V79	In vitro mammalian chromosome aberration test, with and without metabolic activation	cZnO and uZnO (NM-111, NM-110)	Negative
	Someya et al. [33]	Human dental pulp cells (D824 cells)	In vitro mammalian chromosome aberration test, with and without metabolic activation	ZnO	Induced chromosome aberration
	Fraunhofer ITEM [34]	Mouse lymphoma L5178Y	Mammalian cell gene mutation assay	cZnO and uZnO (NM-111, NM-110)	Ambiguous- relevant increase in mutant frequency always linked to cytotoxicity for mouse lymphoma L5178Y
	Corradi et al. [35]	A549 cells	In vitro mammalian cell micronucleus test without metabolic activation	ZnO	Negative, induced genotoxicity appeared to be an indirect consequence of high levels of cytotoxicity
	Demir et al. [36]	TA1535, TA1537, WP2uvrA, RS112	Ames test and DEL assay, with and without metabolic activation	nano ZnO	Up to 640 μg/mL, no genotoxicity in any of the test systems
	Singh et al. (Review) [37]	In vitro*:* various human cell lines In vivo*:* mouse and rat (oral)	Various in vitro and in vivo assays	nano ZnO	In vitro: oxidative stress-related genotoxicity, genotoxicity driven by Zn ion release of ZnO nanoparticles, (oxidative) DNA damage due to various reasons In vivo*:* ROS-induced genotoxicity, DNA damage, apoptosis and lung genotoxicity mediated by oxidative stress induction
	Fraunhofer ITEM [14]	Rat (inhalation)	In vivo Mammalian Erythrocyte Micronucleus Test (OECD TG 474)	cZnO and uZnO (NM-111, NM-110) and µZnO (NM-113)	Negative
	BASF SE [38]	Mouse (oral)	In vivo Mammalian Erythrocyte Micronucleus Test (OECD TG 474)	cZnO (NM-111)	Negative

Various ZnO (nano)forms have already been tested in repeated dose toxicity studies by oral and inhalation exposure. Inflammatory effects at the portal of entry are the predominant observations after repeated exposure to (nano) ZnO [14–26]. After oral exposure also systemic effects were observed [20–22]. Also in sub-chronic and subacute inhalation studies with various ZnO nanoforms, rapid clearance from the lung was observed, suggesting a swift dissolution process upon deposition within the respiratory tract [14–16]. This is in line with abiotic dissolution data, showing a fast dissolution at low pH [39–41]. These data suggest that the toxicity of different ZnO forms is mainly driven by released Zn cations.

The reproduction and developmental toxicity of ZnO was examined in various studies, many of them of exploratory nature rather than according to OECD standards. In some, but not all those studies, high doses caused parental toxicity and effects on reproduction after oral exposure [42–49], whereas these effects were not seen in other studies [50, 51].

The genotoxicity of different ZnO particles including nanoforms were extensively tested [37, 39]. The various investigations resulted in a mixed outcome. Overall, ZnO and its nanoforms (uncoated and coated) did not induce mutations in bacterial assays and in in vitro mutagenicity tests in mammalian cells [30–32, 34–36]. Ambiguous results occurred only at concentrations which also caused pronounced cytotoxicity. At these cytotoxic concentrations, indications for clastogenic effects were observed in the tk-assay [32, 34], which could not be confirmed by a number of chromosomal aberration and micronucleus assays. In addition, no increase in the micronucleus frequency was observed in the peripheral blood cells of rats after subacute inhalation exposure with coated and uncoated ZnO nanoforms. [14, 15, 18, 31, 32, 34, 35].

**Fig. 1:**
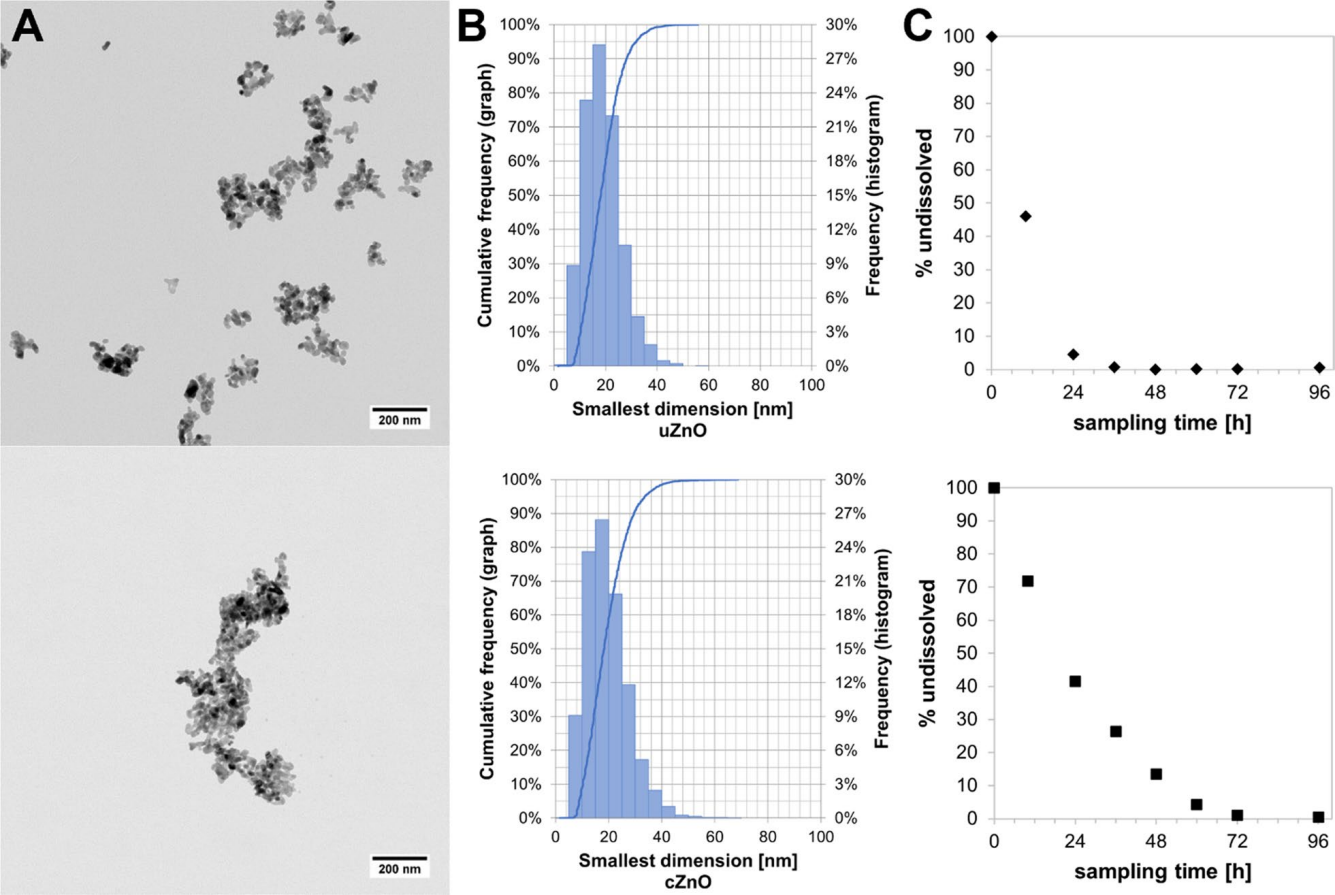
Test item characterization. **A** Transmission electron microscopic (TEM) images, **B** Size distribution of the constituent particles by TEM, **C** Dissolution kinetics in the abiotic CFS method of the test substances, uZnO (top) and cZnO (bottom). Scale bar 200 nm

In general, discrepancies between previous toxicological studies, such as repeated dose (inhalation or oral) studies, reprotoxic studies and genotoxicity studies, are discussed to be attributed to variations in particle size, surface modification, and deposition kinetics. Additionally, differences in exposure modes, aerosol generation, and test concentrations could also contribute to the conflicting findings. Most of the studies were performed with nano ZnO particles from the JRC Nanomaterials Repository (coated nano ZnO, NM-111, and uncoated nano ZnO, NM-110) and showed similar results which could be attributed to Zn ions. However, new ZnO nanoforms vary in both size and coating and it remains unclear whether they show similar toxicity. To address this, the physicochemical properties of various ZnO nanoforms were characterized in a preliminary study (for details see Supplementary Information (SI), Additional file 1 and Table S1). Based on its results, two ZnO nanoforms, one coated (cZnO) and one uncoated (uZnO), with different dissolution kinetics in an abiotic dynamic dissolution assay (see Fig. 1), were selected for the current study. These two nano materials represent the boundaries of different ZnO nanoforms in terms of dissolution: same size with different surfaces (uncoated vs. coated). Furthermore, one uncoated microscale ZnO (µZnO) was tested representing the same surface with a different size (uZnO nanoform vs. µZnO). The two ZnO nanoforms were examined for local and systemic toxicity as well as reproductive and developmental (neuro)toxicity and genotoxicity after inhalation exposure. Soluble zinc sulfate monohydrate (ZnSO_4_*H_2_O, abbreviated as ZnSO_4_) and µZnO were tested in parallel to understand whether observed effects of nanoforms were resembling Zn ion or size-related particle toxicity.

## Material and methods

The present studies were conducted according to the OECD Principles of Good Laboratory Practice [52], which principally meet the United States Environmental Protection Agency Good Laboratory Practice Standards [40 CFR Part 160 (FIFRA) and Part 792 (TSCA)]. The study was conducted referring to OECD Guideline 413 [53], 421 [54], 424 [55], 426 [56], and 489 [57].

### Test materials and characterization

To examine the impact of coating, two ZnO nanoforms (CAS-No.: 1314-13-2), which are available on the European market, with similar sizes and manufactured from identical starting material, were tested: uncoated ZnO nanoform (purity > 98%, specific surface area (BET) 10 m^2^/g), hydrophobic coated ZnO nanoform (purity 94%, BET 10 m^2^/g). In a preliminary study, the characterization of the size distribution of the constituent particles, and their aspect ratio, was performed by Transmission Electron Microscopy (TEM) in accordance with the JRC NanoDefine Manual [58]. For additional details on TEM testing see SI, Additional file 1.

The dissolution behavior in lysosomal pH 4.5 conditions was studied by continuous flow system (CFS) in the lysosomal medium PSF, developed by NIOSH [59]. The method is compliant with a draft OECD guidance (WPMN Task 1.5) and was previously validated against in vivo clearance [41, 60, 61]. Additional details on dissolution testing are given in SI, Additional file 1.

Further, a µZnO material (purity > 99%, BET 4.5 m^2^/g) was tested to assess the potential particle-size triggered difference in toxicity and a soluble salt ZnSO_4_ (CAS-No.: 7446-19-7, Zn content 36.2% weight-%) was tested at equal molarity to ZnO to demonstrate the effects caused by Zn ions. Zinc chloride could not be used due to its strong hygroscopic and highly irritant properties. Instead, ZnSO_4_*H_2_O was used because it is one of the common forms of ZnSO_4_, which is readily soluble in water.

### Animals

All protocols followed the OECD guidelines. The studies were performed in an AAALAC-approved laboratory in accordance with the German Animal Welfare Act and the effective European Council Directive (approval No. 23 177-07/G17-3-063). Male (about 7–8 weeks of age) and nonpregnant nulliparous female (7 weeks of age, 90-day study only) Wistar (strain Crl:WI (Han)) rats were obtained from Charles River Laboratories (Sandhofer Weg, Sulzfeld, Germany) and were allowed free access to mouse and rat maintenance diet (Granovit AG, Kaiseraugst, Switzerland) and water before and after exposure. The animals were housed in polycarbonate cages before and after exposure in accommodation maintained at 22 ± 2 °C, with a relative humidity of 55 ± 10%, a light/dark cycle of 06.00–18.00 h light and 18.00–06.00 h dark and were allowed to acclimatize to these conditions for approximately two weeks before the start of the study.

### Exposure

In terms of exposure, there is a preference for nose-only exposure over whole-body exposure due to the potential for cross-exposure through dermal and oral routes in whole-body chambers, primarily through grooming behavior. However, in the particular studies in this paper, whole-body exposure was chosen as the only viable option for exposing the pups. To maintain consistency throughout the study, the decision was made to continue using the whole-body chambers rather than switching exposure modes. Detailed considerations are elucidated in the general consideration of the discussion section.

During exposure, rats were kept in wired cages located in a glass-steel inhalation chamber (whole-body). From post-natal day (PND) 4 to PND 22, dams were exposed with their litter in perforated polycarbonate cages, with a small amount of low dust bedding. During exposure, food and water were withdrawn, but dams with pups received hydrogel pads from PND 14–16 onward until PND 21. Negative pressure was maintained inside the inhalation chamber to avoid contamination of the laboratory. The exposure systems were kept under exhaust hoods in an air-conditioned room. Detailed information is provided in SI, Additional file 2.

### Aerosol generation and characterization

**Fig. 2:**
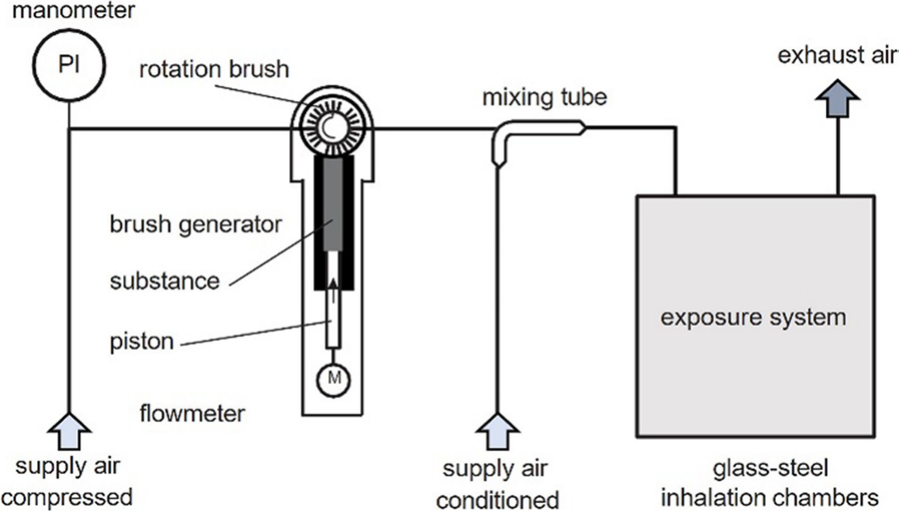
Schematic view of the dust generation and exposure system

Dust aerosols were generated with a brush generator (BASF SE) with 15–5000 mg test substance per hour (adjusted by altering the speed of the feed piston and rotation speed of the brush) mixed with compressed air inside a mixing tube, diluted with conditioned air (activated charcoal-filtered air, 22 ± 2 °C, 50 ± 20% relative humidity) and passed into the inhalation chamber (see Fig. 2). Inhalation exposures were conducted in 1.4 m^3^ stainless steel chambers (BASF SE). During the exposures, particle concentrations were continuously monitored using scattered light photometers (VisGuard; Sigrist-Photometer AG, Switzerland), and the time averaged concentration was recorded three times per group over the 6-h exposure period by gravimetric measurements. The particle size distribution of the aerosol was measured twice per week per group (excluding the control chambers) using an 8-stage cascade impactor (Marple 298, New Star Environmental, Inc., Roswell, Georgia 30075, USA). Moreover, scanning mobility particle size measurement (SMPS; Grimm Aerosol Technik GmbH & Co KG, Ainring, Germany) was performed.

### Range-finding study

To select test concentrations for the main studies, a 14-day range-finding study was performed. The endpoints and examination time points of this range-finding study were based on the OECD test guideline “28-Day (Subacute) Inhalation Toxicity Study” (OECD TG 412, 2018). Groups of 5 male Wistar rats were whole-body exposed to uZnO and cZnO dust aerosol for 6 h per day on five days per week (total of 10 exposures) at target concentrations of 0, 12, and 24 mg/m^3^. On the day after last exposure, bronchoalveolar lavage, hematology as well as histological examination of the respiratory tract were performed.

### 90-day study

**Fig. 3:**
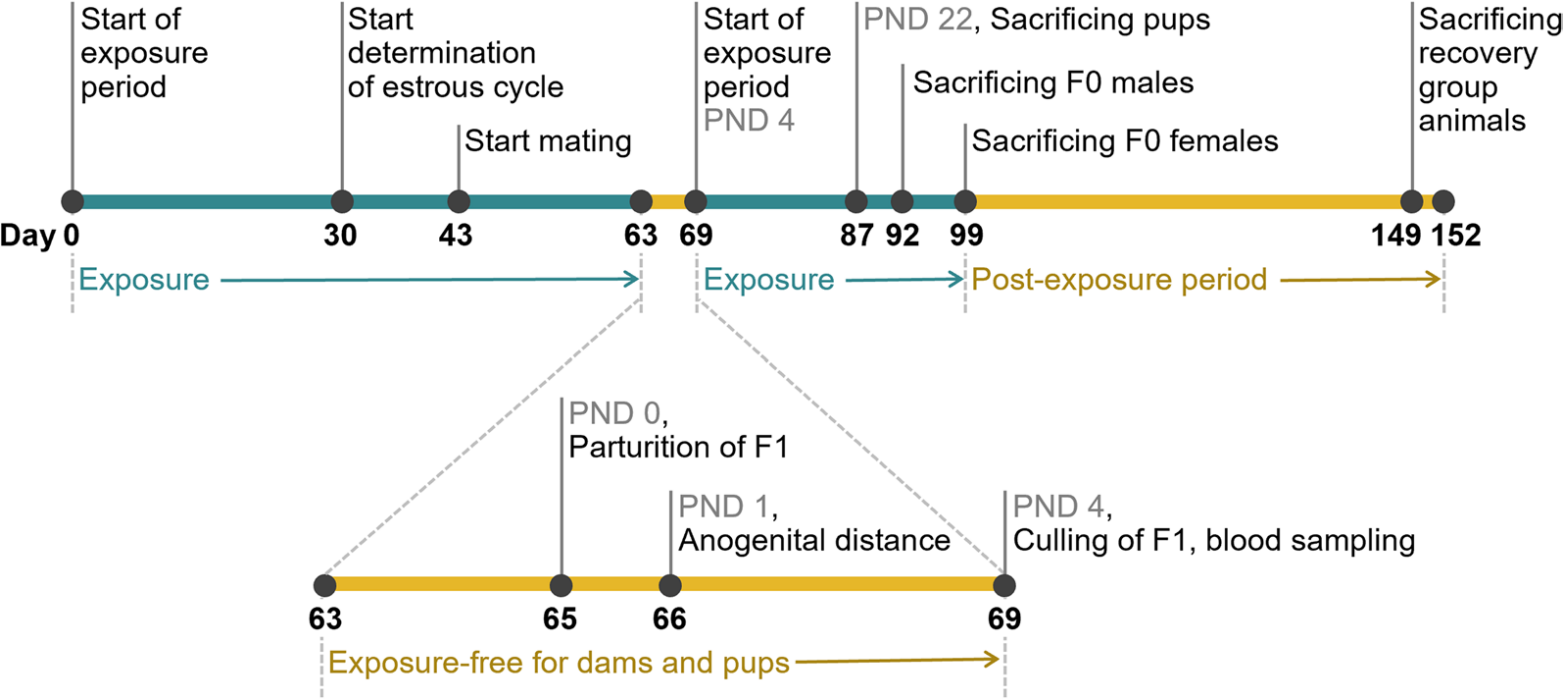
Timeline of 90-day inhalation exposure followed by 8-week post-exposure observation period

The concentrations were selected based on the publicly available data, unpublished 90-day inhalation study by Fraunhofer ITEM [15] and the current range-finding study. Due to the impaired body weight development, reduced food consumption, minimal to severely degeneration of the olfactory epithelium, severely increased absolute and relative lung weight, and severely increased lavage parameters in the current range-finding study (details see results section), the following concentrations were chosen for the 90-day inhalation study: uZnO and cZnO at 0.5, 2, and 10 mg/m^3^, µZnO at 10 mg/m^3^, ZnSO_4_ at 22 mg/m^3^ (Zn^2+^ equimolar 10 mg/m^3^ ZnO). The study design incorporated the elements of OECD TG 413, 421, 424, and 426. The details of the exposure, mating, gestation, lactation schedules are outlined in Fig. 3 and SI, Additional file 2, Table S3. Groups of male and female Wistar rats were whole-body exposed to aerosols of ZnO nano materials, as well as µZnO and ZnSO_4_ for 6 h a day, for 13 weeks. The animals were exposed for 43 days before the mating period of max. 14 days. After the mating period, the exposure of all male F0 animals were continued until they were exposed for a total of minimum 90 days, whereas the female F0 animals were exposed further until gestation day (GD) 19. To allow them to deliver and rear their pups (F1 generation), they were not exposed from GD 20 to PND 3. From PND 4 through to PND 21, dams were exposed with their pups.

This comprehensive study aimed to investigate the toxicity of a substance, focusing on both local and systemic effects according to OECD TG 413. Daily clinical examinations, body weights, food consumption, ophthalmology, detailed clinical observation, functional observation battery (FOB) and motor activity (MA) were recorded. The respiratory tract and remote organs were examined by histopathology, along with bronchoalveolar lavage, hematology, and clinical chemistry of the blood. Additionally, the study evaluated the integrity and performance of the male and female reproductive systems including gonadal function, estrous cycles, mating behavior, conception, gestation, parturition, lactation, and weaning. Histological examinations of the respiratory tract and remote organs were assessed by histopathology, the growth and development of the offspring, including developmental neurotoxicity of offspring on PND 22 were also examined according to OECD TG 421 and OECD TG 426. To assess potential impacts on thyroid function, the levels of thyroid hormones (T4 and TSH) were analyzed in both the parental male animals and the male and female pups on PND 22.

Recovery groups of male and female animals were included after an exposure period of about 90 days, these animals were kept for an additional period of approximately 60 days without exposure.

An overview of the examined animal subsets is given in SI (Additional file 2, Table S4). As part of the main study with 90-day inhalation exposure, the examinations for reproductive toxicity, neurotoxicity and developmental toxicity, pathology and neuropathology are described in detail in the following sections.

### Examinations for reproductive toxicity

After 43 days of premating period, F0 generation parental animals were mated in a 1:1 ratio for maximum two weeks or until there was evidence for copulation. A vaginal smear for sperm was prepared once daily and the first positive test referred to GD 0. Throughout the pre-mating and mating period, the estrous cycle of parental females was monitored. Mating success as well as gestation and parturition outcome were recorded and from these data corresponding indices were calculated which facilitate the assessment of reproductive performance and pre-postnatal development.

Offspring was examined on the day of birth (PND 0) and standardized to evenly distributed 10 pups per litter, preferably 5 male and 5 female pups per litter (PND 4). Moreover, pups were allocated to different examination groups before weaning (PND 21). For each substance, groups of 10 male and 10 female pups were selected for determination of thyroid hormone concentrations on PND 22, perfusion fixation for neuropathological examinations on PND 22, as well as open field observations (OFO) (PND 13 and 21) and MA (PND 13, 17, and 21). Histopathological examination as well as organ burden determination has been performed on 5 animals per sex and substance on PND 22.

Viability of the pups was checked at least once daily, the percentage of pups dying during the lactation period was determined and corresponding indices were calculated. Clinical observation of the pups was performed daily. The body weight was recorded once weekly and before standardization of the litters. The anogenital distance (distance from the center of the anal opening to the base of the genital tubercle) was determined on PND 1 using a measuring ocular and the anogenital index was calculated (anogenital distance [mm]/cubic root of pup weight [g]). Number of nipples/areolas were counted in F1 male pups on PND 13 and PND 20. On PND 4, surplus pups and on PND 22, selected pups (1 male and female/litter) were sacrificed under isoflurane anesthesia by decapitation to sample blood for determination of thyroid hormone concentrations. Sacrificed pups were macroscopically examined for any abnormalities.

### Examinations for neurotoxicity and developmental toxicity

Detailed clinical observations in a standard arena (50 × 37.5 cm with a lateral border of 25 cm) were recorded in all parental and recovery group animals prior to exposure period and once during the first two weeks of the exposure period (abnormal behavior during handling, fur, skin, posture, salivation, respiration, activity/arousal level, tremors, convulsions, abnormal movements, gait impairment, lacrimation, palpebral closure, exophthalmos, feces appearance/consistency, urine, pupil size).

FOB was performed in 10 adult animals/sex/group at the end of the exposure period (no inhalation exposure on this day). The examinations included home cage and OFO, sensorimotor as well as reflex tests in a randomized order. Feed but not water was withdrawn before the examinations. At least 30 min before home cage observations, animals were transferred from group to individual cages (floor area about 800 cm^2^). Animals were observed in closed home cages without any disturbances such as noise or touching the racks with a special focus on posture, tremor, convulsions, abnormal movements, as well as gait. For OFO, animals were transferred to a standard arena (50 × 50 cm for adults and 50 × 37.5 cm for pups with a lateral border of 25 cm) and observed for 2 min to check on behavior on removal from cage, fur, skin, salivation, nasal discharge, lacrimation, eyes/pupil size, posture, palpebral closure, respiration, tremors, convulsions, abnormal movements/stereotypies, gait abnormalities, activity/arousal level, as well as feces appearance/ consistency, urine amount/color, number of rearing (adults only) within the test period.

Thereafter, sensorimotor, and reflex tests were performed on parental animals to examine approach and touch response, vision (“visual placing response”), pupillary and pinna reflex, audition (“startle response”), coordination of movements (“righting response”), behavior during handling, vocalization, pain perception (“tail pinch”), grip strength of limbs, landing foot-splay test.

MA was determined in polycarbonate cages each with 18 infrared beams to measure horizontal (locomotor) and vertical (rearing) movement (TSE Systems GmbH, Bad Homburg, Germany). Testing was conducted in the afternoon, with no feed or water. For each animal, the distance traveled and the number of rearings were quantified over 12 consecutive 5-min periods. For measurement of MA in pups on PND 13, 17, and 21, cages were equipped with two sensor rings, the lower ring with 18 light beams and the upper ring (rearing) with 12 light beams.

### Examinations for clinical pathology

In the 90-day study, pathological examinations were performed according to OECD TG 413. Blood samples were taken from male and female parental animals (fasted overnight) by retroorbital venous plexus puncture under isoflurane anesthesia on the day after the last exposure as well as at the end of the post-exposure observation period. Hematological and clinical chemical parameters were determined according to OECD TG 413 (for details see SI, Additional file 2). Determination of thyroid hormone concentrations were performed according to OECD TG 421 by TSH radioimmunoassay (tat TSH RIA, Izotop, Budapest, Hungary) on a gamma counter (LB 2111, Berthold, Germany) and T4 ELISA (T4 ELISA, DRG instruments GmbH, Marburg, Germany) on a MTP reader (Sunrise MTP-reader, Tecan AG, Maennedorf, Switzerland). BALF was obtained in the range-finding study, 14-day and 90-day study, after sacrificing animals by exsanguination under Narcoren^®^ anesthesia and flushing lungs with two instillations of physiological saline to determine total protein concentration, total cell count, differential cell count as well as activities of the BALF enzymes as described by Ma-Hock et al. in 2009 [62].

### Examinations for pathology and neuropathology

Animals were necropsied after exsanguination under pentobarbital anesthesia. In the 90-day study, selected organs of animals were weighed according to OECD TG 413. Uteri of F0 parental animals were examined for implantation sites. All organs and tissues listed in OECD TG 413 were fixed in 4% buffered formaldehyde or modified Davidson’s solution (for eyes with optic nerves, ovaries, epididymis, testes), paraffin embedded, sectioned, and stained with hematoxylin–eosin for histopathological light-microscopy examinations. In F0 animals, full histopathological examinations were performed on animals of the control and high concentration group. Ovaries, epididymides, and testes of animals of all concentration levels were examined in all mating pairs suspected of reduced fertility. The respiratory tract was examined in all F0 as well as recovery group animals. In the F1 generation, parts of the liver were fixed in Carnoy’s solution and paraffin embedded for all test groups on PND 22. Lungs were examined in all F1 pups. Additionally, nasal cavity level III was examined in all pups exposed to cZnO due to findings in the highest test concentration.

According to OECD TG 426, neuropathological examinations were performed on pups on PND 22. Therefore, animals were sacrificed by perfusion fixation (SOERENSEN phosphate buffer and 4% buffered formaldehyde solution) under i.p. pentobarbital anesthesia. Final body weight, brain weight and measurements were determined. Selected organs including brain with olfactory bulb, eyes with retina and optic nerve, nose, pituitary gland, and trigeminal ganglia were paraffin embedded, sectioned, and stained with hematoxylin–eosin for neurohistopathology. Neurohistopathological examinations were performed in pups of all test groups, including brain cross sections and brain-associated tissues. Morphometry (Hamamatsu NanoZoomer 2.0 (HT C9600 Series) in all pups of control and high concentration groups comprised the neocortex, hippocampus, and cerebellum. After completion of the histopathological assessments an internal peer review was performed. Scores for histological findings were calculated by multiplying incidences and gradings of each finding and dividing by the number of animals.

### Examination for organ burden

To assess organ burden of lungs, liver, heart, brain, and the olfactory bulb, fresh tissue samples were taken to perform inductively coupled plasma optical emission spectroscopy (ICP-OES) in control and high concentration group animals. To quantify the Zn burden per organ, approximately 100 mg of each organ were weighed into a beaker and treated with 10 mL H_2_SO_4_. Afterwards, 3 mL HNO_3_ was added and the mixture was heated. After adding 5 mL of an acidic mixture (nitric acid, sulfuric acid, perchloric acid, 1:1:1, v/v/v), it was heated again until complete dissolution. The mineral acids were evaporated via heating and the residue was dissolved and diluted again with HCl and purified water, resulting in a final concentration of 5% HCl (v:v). The final Zn concentrations were determined via external calibration using an ICP-OES (iCAP PRO thermo fisher scientific, Waltham, USA). Sc was used as internal standard.

In the 90-day study, histological sections of the left lung lobes (parental main group male animals, F0) of the control and high concentration group were taken for quantitative bioimaging (detailed tissue preparation see *Pathology and Neuropathology*). Lung sections with a thickness of 7 µm were applied on microscopic slides, and deparaffinized prior to analysis. A 193 nm ArF excimer laser ablation system (NWR193, Elemental Scientific Lasers, Bozeman, USA) equipped with a two-volume cell (TwoVol2 Ablation Cell, Elemental Scientific Lasers, Bozeman, USA) was coupled to an ICP-TQMS (8900 ICP-MS Triple Quad, Agilent Technologies). To quantify Zn and iron (Fe) in the tissue sections, external calibration using gelatin standards was applied. Therefore, gelatin was spiked with different Zn and Fe concentrations and cut into sections with a thickness of 7 µm. For low-resolution analysis, lung sections and gelatin sections were ablated with a spot size of 40 μm and a scan speed of 120 μm s^−1^. Imaging analysis with a spot size of 10 µm and a scan speed of 30 μm s^−1^ was applied for high-resolution analysis. The ions ^31^P^16^O^+^, ^56^Fe^16^O^+^, ^66^Zn^+^, and ^68^Zn^+^ were detected. The laser fluence was optimized to ensure quantitative ablation, which was controlled using light microscopy.

### Comet assay after 14-day inhalation exposure

The comet assay after inhalation exposure was performed to assess potential genotoxicity of the test substances. The generation and characterization of the test atmosphere were conducted in accordance with the OECD guidance Document No. 39 and OECD TG 412, and the evaluation of the samples was based on OECD TG 489. Based on the results of the range-finding study, the uZnO and cZnO were tested at 0.5, 2, and 8 mg/m^3^, µZnO at 8 mg/m^3^, ZnSO_4_ at 18 mg/m^3^ (Zn^2+^ equimolar 8 mg/m^3^ ZnO). Inflammatory response induces DNA damage causing mutations and cytotoxicity. To avoid any inflammation driven artifacts in the performed comet assay, the highest test concentration level was set to 8 mg/m^3^ ZnO (compared to 10 mg/m^3^ in the current 90-day inhalation study as described below).

Groups of five male Wistar rats were exposed whole-body to the indicated concentrations of each test substance for a 6-h period per day for 14 consecutive days via inhalation route (14 exposures). In addition, ethylmethane sulfonate (EMS) was used as a positive control and applied orally at a single time point at a dose rate of 300 mg/kg body weight (b.w.). On the day after last exposure bronchoalveolar lavage as well as histological examination of the respiratory tract were performed.

To ensure comparability and minimize the time between the end of exposure and sacrificing (about 10 min), each group's exposure was sequentially started, and the animals were sacrificed over three days. Tissues for the comet assay, including bone marrow, left lung lobe, liver, and nasal mucosa, were collected, processed, and analyzed according to OECD TG 489. All comet samples were collected from each animal within 20 min after exsanguination. Collected samples were rinsed or flushed as necessary with room temperature mincing buffer (Ca^2+^ and Mg^2+^ free Hanks Balanced Salt Solution with 10% v/v DMSO and 20 mM Na_2_-EDTA, pH 7.6) and submerged in fresh room temperature mincing buffer before they were placed on ice and processed at Helix3 Inc., Morrisville NC, USA. To obtain single cell suspensions of the lung and liver, approximately 0.5–1 cm^2^ of each tissue was quickly minced in the same mincing buffer in which it was maintained on ice. The bone marrow samples were collected by flushing 1 mL of room temperature mincing buffer through one femur and aspirating the sample to generate a homogenous cell suspension. The nasal mucosa samples were collected by gently scraping the interior of the nasal cavity into 0.5 mL of room temperature mincing buffer and aspirating the sample to generate a homogenous suspension. The cell suspensions were maintained on ice throughout processing. All slide preparation steps were conducted at a relative humidity of ≤ 60%. For each comet slide prepared, an aliquot of cell suspension was mixed with 37 ± 2 °C low melting point agarose (0.5% w/v in PBS), layered onto microscope slides precoated with normal melting point agarose, and maintained cold until the agarose solidified. The coverslip was then removed to add another layer of low melting point agarose, the coverslip replaced, and the slides were maintained cold until all slides for the day had been prepared. After all the daily slides were prepared, they were placed in cold working high salt lysing solution (2.5 M NaCl, 100 mM Na_2_-EDTA, 10 mM Trizma base, 1% v/v Triton X-100, and 10% v/v DMSO, pH 10.0) and maintained cold. Slides were placed in lysing solution in random order with < 10 min between the first and last slide to minimize procedural bias and ensure that the lysis time for all study slides was maintained as consistent as possible. For electrophoresis, 2 replicate comet slides per tissue were lysed overnight before they were rinsed with 0.4 M Tris buffer and placed in a recirculating gel box (Fisher FB-SBR-2025). The slide distribution with each gel box was randomized and balanced across dose groups and replicate sets of slides as much as possible to mitigate any influence of gel box/power supply differences, slide position, and/or dose group processing order. The gel boxes were placed in a 1–10 °C cold room and freshly prepared alkaline electrophoresis buffer (0.3N NaOH and 1 mM Na_2_-EDTA, pH > 13) was added until the slides were submerged to unwind and denature the DNA for 20 ± 1 min. The 10N NaOH and 200 mM Na_2_-EDTA stock solutions used to prepare the fresh electrophoresis buffer was prepared < 2 weeks before use and maintained sealed in an airtight container to ensure the stability and integrity of the solutions. After unwinding, the slides were electrophoresed with passive buffer recirculation at 1–10 °C for 40 min at 0.7 V/cm and at a starting current of 300 ± 10 mA. Afterwards, comet slides were neutralized with 0.4 M Tris buffer, rinsed in ethanol, and air dried. To address cytotoxicity, a low molecular weight (LMW) DNA diffusion assay was performed [63, 64]. One replicate comet slide per sample was removed from lysis after 1 h, neutralized with 0.4 M Tris buffer, rinsed with ethanol, and air dried without electrophoresis.

Comet slides were stained with SYBR Gold™ stain and 150 cells per sample (75 cells per replicate slide) were scored quantitatively using the Komet7© Image Analysis System (Andor Technology, Northern Ireland) with a CoolLED pE-300 light source and a 14 bit EMCCD Luca-R camer (Andor Technology, Northern Ireland). Slides were scored without knowledge of the sample dose group and comets were scored in an unbiased manner by starting scorning near the center of the slide and indiscriminately scoring the first 75 comets that came into view while moving the microscope stage. The only comets excluded from scoring were cells the image analysis system could not accurately measure due to an indiscernible head (i.e., “ghosts”) or overlapping cells or debris. The criteria indicated in OECD TG 489 were applied for the validity assessment of the study part.

### Statistical analysis and modelling

Statistical significance was defined as *p* ≤ 0.05 compared with the control group. Control groups were compared with dose groups. Statistical tests are indicated in a footnote below the respective data tables. MPPD modeling v 3.04 (Applied Research Associates, Inc., Albuquerque, USA) was used with the following parameters: asymmetric Sprague Dawley rat, body weight of 450 g, whole-body exposure, density of 2 g/mL (due to agglomeration of the particles), and an aspect ratio of 1.

## Results

### Characterization of ZnO nanomaterials

The primary particle size of uZnO was in the median 18.2 nm, while cZnO was 18.3 nm in the median, respectively. Both materials were spheroidal with an aspect ratio of 1.3, hence assigned to the spheroidal shape category (Fig. 1A). The characterization of the particle size distribution (PSD) was calculated from the images by automated image analysis with high statistical relevance (Fig. 1B) [65]. In Fig. 1C, dissolution behavior of the selected ZnO nanomaterials (uZnO and cZnO) are presented. For additional details see SI, Additional file 1: Table S2.

### Results of the range-finding study

*Uncoated ZnO nanoform* Characterized parameters of the test atmosphere are given in Table 2. No adverse effects on body weight and food consumption or in blood analysis were observed. In bronchoalveolar lavage fluid (BALF), total cell count, neutrophil-, monocyte-, and lymphocyte count increased significantly, while relative macrophage counts decreased. Protein concentration and enzyme activities, such as lactate dehydrogenase (LDH) and alkaline phosphatase (ALP), also increased. During necropsy, absolute (135%) and relative lung weights (138%) were increased after exposure to the high concentration (20.7 mg/m^3^). Histological examinations showed slight infiltration of neutrophilic granulocytes and moderate alveolar histiocytes in the lungs of all animals exposed to the high concentration. Moreover, minimal to moderate degeneration and/or regeneration of the olfactory epithelium in the nasal cavity was observed in all animals exposed to the high concentration. All levels of the nasal cavity were affected, with level IV showing the most severe finding. The same findings were still observed after exposure to the low concentration (10.9 mg/m^3^), yet with lower incidence and less severity (for detailed data see SI, Additional file 3: Tables S5–S8). At the low concentration, absolute (113%) and relative lung weights (118%) were still increased, although not statistically significant.

**Table 2:** Characterization of the test atmosphere

Parameter range-finding study	uZnO		cZnO		µZnO	ZnSO_4_
Target Concentration (mg/m^**3**^)	12	24	12	24	–	–
Measured Concentration ± SD (mean) (mg/m^**3**^)	10.9 ± 0.41	20.7 ± 0.89	10.8 ± 0.37	21.3 ± 1.02	–	–
MMAD (µm)/GSD (2 measurements)	1.90/2.03 1.79/2.04	1.85/2.02 1.77/2.17	1.99/1.99 1.92/2.17	1.77/1.97 1.79/2.26	–	–
SMPS [nm] ± SD (median)	297 ± 41	228 ± 51	231 ± 20	193 ± 11	–	–

Parameter 14d study (comet assay)	uZnO			cZnO			µZnO	ZnSO_4_
Target Concentration (mg/m^**3**^)	0.5	2	8	0.5	2	8	8	18
Measured Concentration ± SD (mean) (mg/m^**3**^)	0.5 ± 0.06	2.1 ± 0.15	7.9 ± 0.53	0.5 ± 0.12	2.0 ± 0.41	7.9 ± 0.97	7.6 ± 0.88	17.8 ± 0.86
MMAD (µm)/GSD (3 measurements)	1.95/1.98 1.88/1.93 1.32/2.48	2.20/2.27 1.85/2.41 2.00/2.24	2.03/2.01 1.53/1.97 1.63/2.55	2.21/2.16 1.71/2.03 1.83/2.02	1.61/2.19 1.63/2.32 2.05/2.08	1.48/1.95 1.75/1.97 1.72/2.01	0.92/1.93 0.83/1.91 1.02/2.22	2.20/2.30 2.47/1.88 2.80/1.97
SMPS [nm] ± SD (median)	215 ± 24	344 ± 25	402 ± 30	222 ± 78	285 ± 15	303 ± 26	227 ± 13	324 ± 8

Parameter 90d study	uZnO			cZnO			µZnO	ZnSO_4_
Target Concentration (mg/m^**3**^)	0.5	2	10	0.5	2	10	10	22
Measured Concentration ± SD (mean) (mg/m^**3**^)	0.5 ± 0.10	2.0 ± 0.20	10.0 ± 1.23	0.5 ± 0.16	2.0 ± 0.20	10.1 ± 0.96	9.7 ± 1.47	21.9 ± 1.30
MMAD (µm)/GSD (median of 14 measurements)	1.34/2.52	1.4/2.34	1.19/2.34	1.73/2.26	1.65/2.14	0.97/2.32	0.82/2.04	2.25/2.04
SMPS (nm) ± SD (median)	213 ± 75	305 ± 113	222 ± 79	290 ± 48	326 ± 51	228 ± 6	216 ± 11	324 ± 9

*MMAD* mass median aerodynamic diameter, *GSD* geometric standard deviation, *SMPS* scanning mobility particle sizer

*Coated ZnO nanoform* Characterized parameters of the test atmosphere are given in Table 2. Body weight gain and food consumption were reduced in animals exposed to the high concentration in comparison to the control group. Increases of BALF parameters were within the range of those observed with uZnO, at the respective concentrations. The absolute and relative lung weights were increased (141% and 154% at 21.3 mg/m^3^, respectively). The histological findings in the lungs and nasal cavities were largely consistent with the findings after exposure to the uZnO, some individuals, however, had more severe findings (for detailed data see SI, Additional file 3: Tables S5–S8). At the low concentration of 10.8 mg/m^3^, absolute (114%) and relative lung weights (119%) were still significantly increased, albeit less severe than at 21.3 mg/m^3^.

*Concentration selection for the 90-day study* Considering the severe effects observed in the respiratory tract, impaired body weight development, and food consumption, it was determined that the high concentration tested in the range-finding study would not be tolerable in the 90-day study. Instead, a concentration of 10 mg/m^3^ was selected and considered to be still sufficient to cause toxicity in that study.

### Sub-chronic study

*Characterization of the atmosphere* The obtained atmospheric concentrations were close to the target concentrations and are displayed together with particle size distribution data in Table 2. The real time surveillance photometer data proved that the dust aerosol concentrations were maintained throughout the duration of the study; air flows, mean chamber temperature (20.3–23.9 °C) and mean relative humidity (42.3–62.8%) were in the desired range. The cascade impactor measurements showed a high fraction of respirable particles (> 70%). The scanning mobility particle sizer (SMPS) showed that the test articles agglomerated in the test atmosphere with median agglomerate diameters of 213–326 nm.

*Clinical signs, body weights, hematology and clinical chemistry including T4 and TSH* There were no substance-related clinical signs of toxicity in male and female animals exposed to any of the ZnO forms. Several male and female animals exposed to ZnSO_4_ showed salivation, indication irritative effects to the respiratory tract. In male and female animals exposed to uZnO, no substance-related deviations of the body weight development in comparison to the controls was observed. In female animals exposed to cZnO, a slightly lower body weight was observed upon exposure to the high concentration (10 mg/m^3^) during gestation and lactation. The maximum difference was 6.7%; the final mean body weight was only about 5% lower than in the control group. Hence, this effect was considered substance-related but not adverse. Transiently decreased body weight was also observed in males exposed to µZnO, which is considered not adverse. In contrast, the body weight development in male and female animals exposed to ZnSO_4_ was retarded and is considered adverse. Ophthalmological examinations, FOB and MA tests of parental male and female animals did not show any treatment-related findings in animals exposed to any of the test substances.

In hematology, only male animals exposed to high concentration (10 mg/m^3^) of cZnO showed a slight but significant increase of total white blood cell counts, as well as absolute neutrophil and lymphocyte count (SI, Additional file 3: Table S12). These changes were considered substance-related and secondary to the local effects in the respiratory tract. No further hematological changes were observed. Examinations of clinical chemistry parameters did not show any adverse effects. No treatment-related alterations of T4 and TSH levels were observed.

**Fig. 4:**
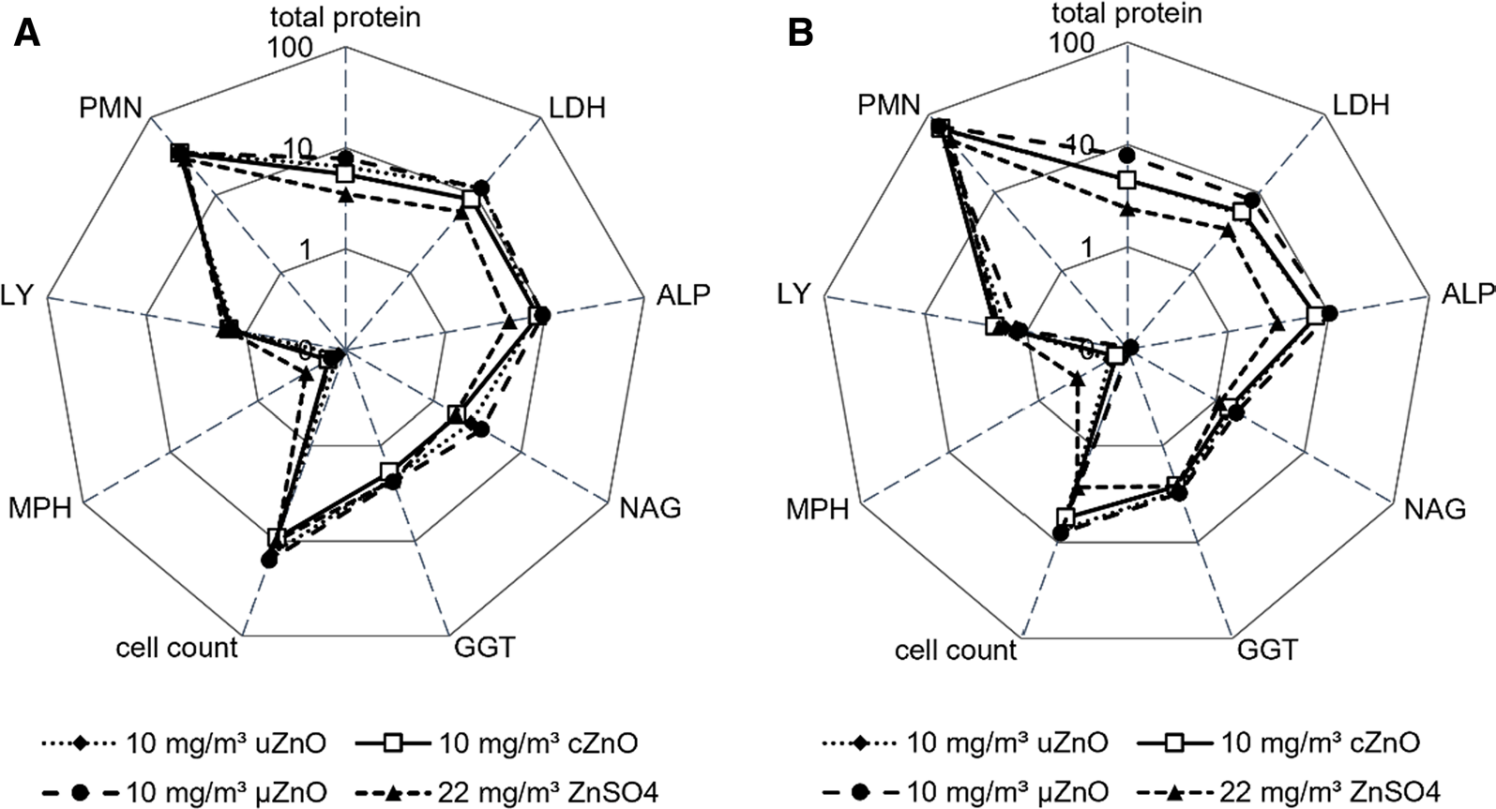
Changes (fold of control) of BALF parameters after 90-day inhalation exposure of adult animals. High concentration groups of **A** male (n = 10) and **B** female (n = 10) animals. Axis in logarithmic scale. Abbreviations: LDH: lactate dehydrogenase, ALP: alkaline phosphatase, NAG: N-acetyl-β-D-glucosaminidase, GGT: γ-glutamyl transferase, MPH: macrophages, LY: lymphocytes, PMN: polymorphonuclear neutrophils

*Lavage* After 90 days exposure to uZnO, cZnO, µZnO, and ZnSO_4_, an increase of the total cell count, due to an increase of neutrophils (PMN) and monocytes, was observed in BALF. Moreover, total protein and bronchoalveolar lavage fluid (BALF) enzyme activities, especially of LDH and ALP, were increased in animals exposed to the high concentrations of all test items. In BALF of rats exposed to the mid concentration of uZnO and cZnO, higher levels of γ-glutamyl transferase (GGT), ALP as well as LDH (male only) were observed (Fig. 4 and SI, Additional file 3: Figure S1, Tables S10–S11). 8 weeks after the end of the exposure to all test substances, all parameters returned to the control level.

*Pathology* An overview of histological findings in adult animals including relative and absolute lung weights is provided in Table 3. The details of incidences and severities of the findings are summarized in the SI, Additional file 3: Tables S13–S14.

**Table 3:** Relative changes of lung weight and histological findings in the respiratory tract of adult animals after 90-day inhalation exposure

		Male animals (n = 10)									Female animals (n = 10)								
		Control	uZnO			cZnO			µZnO	ZnSO_4_	Control	uZnO			cZnO			µZnO	ZnSO_4_
*Concentration (mg/m*^*3*^*)*		0	0.5	2	10	0.5	2	10	10	22	0	0.5	2	10	0.5	2	10	10	22
*Lung weight*	Absolute lung weight	100%	99%	104%	140%**	101%	104%	136%	130%**	125%**	100%	105%	102%	128%**	101%	106%	131%**	137%**	114%**
Changes	Relative lung weight	100%	103%	109%	150%**	107%	105%	143%	141%**	138%**	100%	104%	103%	130%**	102%	106%	137%**	141%**	119%**
*Histological findings:*																			
No. of animals examined		10	10	10	10	10	10	10	10	10	10	10	10	10	10	10	10	10	10
*Lungs*																			
Foamy macrophages, alveolar		0	0	0.5*	2.7**	0	0.5*	2.7**	2.3**	2.5**	0	0	0	2.8**	0	0	2.3**	2.1**	1.4**
Debris, cellular, (multi)focal		0	0	0	2.8**	0	0	2.8**	2.7**	1.3**	0	0	0	1.9**	0	0	1.6**	2.2**	1.1**
Infiltration, neutrophils, (multi)focal		0	0	0.1	2.1**	0	0	1.9**	1.7**	1.1**	0	0	0	1.9**	0	0	1.5**	1.5**	1.1**
Hyperplasia, type II pneumocytes, (multi)focal		0	0	0	1.3**	0	0	0.8**	0.9**	0.8**	0	0	0	1.5**	0	0	1.7**	1.1**	1.1**
*Larynx*																			
Epithelial alteration, (multi)focal		0.1	0.1	0	0.6*	0	0.2	0.2	0.1	0	0	0	0.1	0.4*	0	0.1	0.3	0	0
Metaplasia, squamous, (multi)focal		0	0	0	0	0	0	0	0	1.7**	0	0	0	0	0	0	0	0	1.9**
Inflammatory cell infiltrates (multi)focal		0	0	0	0	0	0	0	0	0.1	0	0	0	0	0	0	0	0	1.3**
Erosion/ulcer		0	0	0	0	0	0	0	0	0	0	0	0	0	0	0	0	0	0.1
*Nasal cavity*																			
Degen./regen. olfactory epithelium (level III)		0	0	0.1	0.5*	0	0.4	0.3	0	1.5**	0	0.1	0	0.9**	0	0.1	0.5*	0	1.5**
Degen./regen. olfactory epithelium (level IV)		0	0	0.1	1.2**	0	0.2	0.6**	0	2.0**	0	0	0	1.0**	0	0.1	1.1**	0	2.0**

For statistical analysis of lung weights, a two-sided Wilcoxon test was performed. For histological findings, Fisher’s exact test (one-sided +) was performed based on incidences of the findings

*p < 0.05; **p < 0.01

**Fig. 5:**
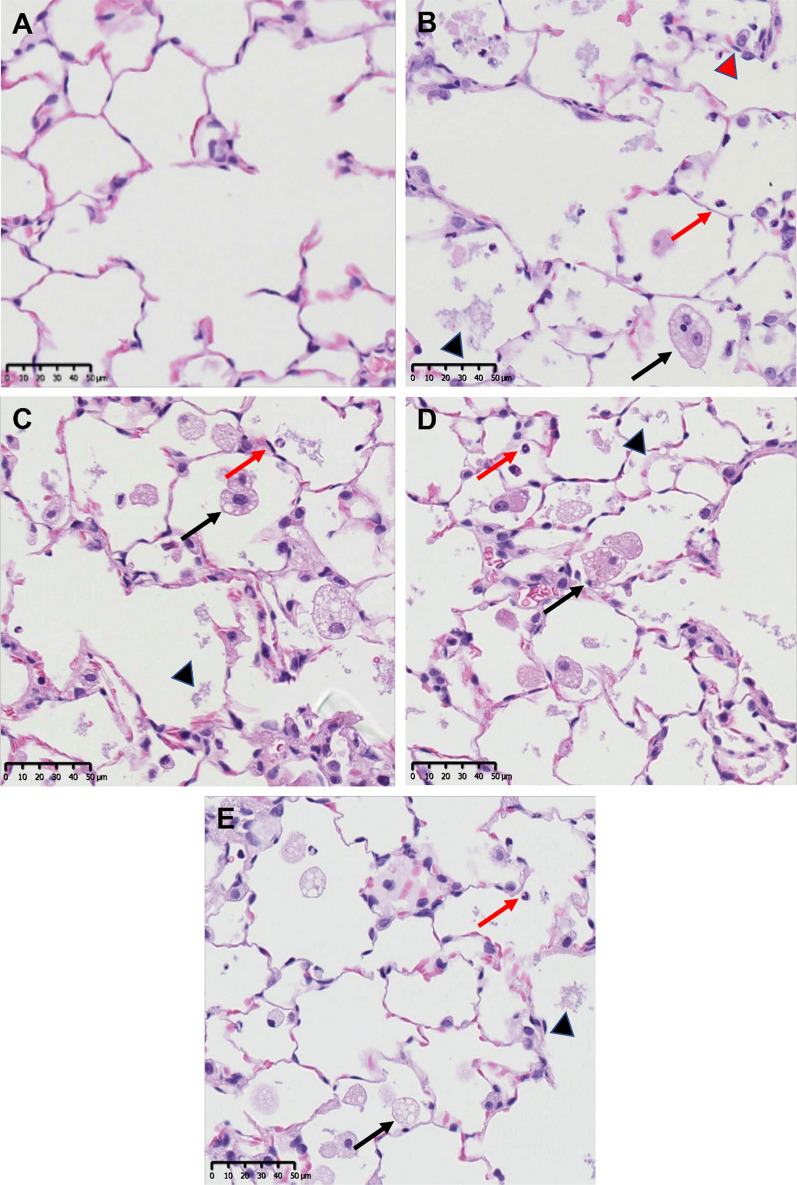
Histologic images of the lungs of male control as well as high dose group animals after 90-day inhalation exposure. **A** control animal, **B** uZnO 10 mg/m^3^ intra-alveolar infiltration of foamy macrophages (black arrow), cellular debris within alveolus (black arrowhead), infiltration of neutrophils (red arrow) and hyperplasia of type II pneumocytes (red arrowhead). **C** cZnO 10 mg/m^3^, **D** µZnO 10 mg/m^3^, **E** ZnSO_4_ 22 mg/m^3^ showed comparable findings as in **B**, hyperplasia of type II pneumocytes not always present. H&E, scale bar 50 µm

Effects were observed in the nasal cavity, larynx, and lungs, but not in other organs. In the lungs of animals exposed to the highest concentrations (10 mg/m^3^ ZnO materials, 22 mg/m^3^ ZnSO_4_) of all test substances an infiltration of neutrophils and multifocal accumulation of foamy macrophages accompanied by an increase of the lung weight and an activation of the draining lymph nodes (mediastinal and tracheobronchial) was observed. Moreover, hyperplasia of type II pneumocytes as well as cellular debris within alveoli was found as result of pulmonary cell damage when exposed to the highest tested concentration of all test substances (Fig. 5). The severity and incidence of these effects greatly decreased in the post-exposure period of 8 weeks but did not completely resolve within. In animals exposed to the mid concentration (2 mg/m^3^) only a few individual foamy macrophages or neutrophilic infiltration were observed. No histological findings were observed in lungs after inhalation of the lowest concentration (0.5 mg/m^3^) of uZnO and cZnO.

**Fig. 6:**
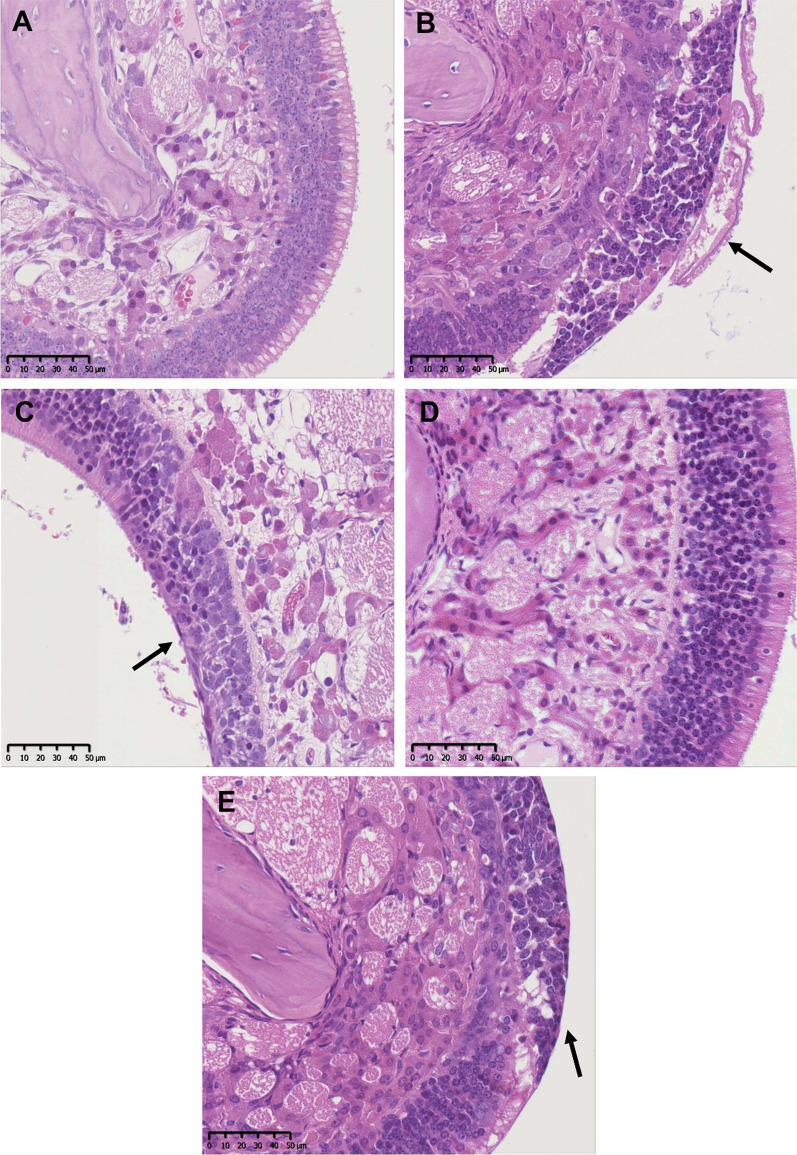
Histologic images of olfactory epithelium (nasal cavity) of male control as well as high dose group animals after 90-day inhalation exposure at the tip of a conchae or at the region of the dorsal meatus. **A** Control animal, **B** uZnO 10 mg/m^3^ (multi)focal degeneration/regeneration of the olfactory epithelium (arrow), **C** cZnO 10 mg/m^3^, and **E** ZnSO_4_ 22 mg/m^3^ showed same findings as in **B** indicated by arrows. **D** Animals treated with µZnO did not show any findings in the nasal cavity. H&E, scale bar 50 µm

In the upper respiratory tract, a loss of olfactory epithelium in the nasal cavity (mainly level IV) was observed in animals exposed to the highest test concentrations of all test materials (slight to minimal with all ZnO materials and moderate with ZnSO_4_) (Fig. 6). A minimal damage of the olfactory epithelium was detected in animals exposed to lower test concentrations of uZnO and cZnO. This reverted to normal for all particulate test substances (uZnO, cZnO, µZnO) after the post-exposure period but sustained after exposure to ZnSO_4_ which had caused the most severe findings directly after the end of the exposure. Moreover, treatment-related epithelial alterations were found in the larynx (mostly level I) of animals exposed to uZnO and cZnO, characterized by an increased number of cell layers and squamous metaplasia. These cells may display slight nuclear polymorphism and cellular atypia. This effect was occasionally observed in control animals as well. Since this alteration fully recovered after the cessation of treatment and does not hinder breathing, it was considered to be treatment-related but not adverse [66]. In animals exposed to ZnSO_4_, inflammatory cell infiltrates, erosion/ulceration of the laryngeal epithelium (one female) and squamous metaplasia at the base of the epiglottis (in all animals) were detected, which remained after the post-exposure period. In rats exposed to µZnO, epithelial alterations were only observed in one male animal.

Comparing the local effects of uZnO, cZnO, and µZnO, the overall findings in lungs, draining lymph nodes, nasal cavity, and changes in lavage parameters were comparable at the tested concentrations. For animals exposed to ZnSO_4_, lower incidences and severity were observed in lungs while both were higher in nasal cavity and larynx. This difference is considered to be related to the larger particle size of ZnSO_4_ than the one of ZnO. Consequently, the deposited dose at the upper respiratory tract was higher, while those deposited in the lungs was lower. However, all observed changes reduced greatly in incidence and severity after the 8-weeks recovery period.

*NOAEC* For both, uZnO and cZnO, the general, systemic NOAEC is above the highest tested concentration of 10 mg/m^3^. Based on effects on BALF and histopathology of the upper and lower respiratory tract, the local NOAEC in male rats is 0.5 mg/m^3^ for both, uZnO and cZnO, and in female rats 0.5 mg/m^3^ for cZnO. Due to findings in the nasal cavity of one of 10 examined female animals exposed to 0.5 mg/m^3^ uZnO, no NOAEC could be determined for female rats. Thus, the lowest observed adverse effect concentration (LOAEC) for female animals within this study was 0.5 mg/m^3^ for uZnO. Local NOAECs for μZnO and ZnSO_4_ were not assigned since these materials were only tested with one high concentration as a size and solubility reference (Table 7).

### Fertility and reproductive performance

Female rats of all test groups had a regular estrous cycle during the last 3 weeks prior to mating, with an average cycle length of 4 days. For all mated female and male animals, copulation was confirmed, (100% mating index) resulting in pregnancies of all or most females (fertility index of 87.5–100%). The mean duration until copulation was between 1.9 and 2.8 days without any relation to the test item. The gestation index was 100% and the mean duration of gestation (21.9–22.4 days) was comparable in all test groups. The number of implantations, post-implantation losses, as well as the rate of liveborn pups (97.9–100% across all test groups) was not affected by the exposure to the test substances. No adverse effects on fertility or reproductive performance of rats exposed to uZnO, cZnO, µZnO, and ZnSO_4_ could be identified (SI, Additional file 3: Table S15). The NOEC of fertility and reproductive performance is 10 mg/m^3^ for both, uZnO and cZnO.

### Developmental toxicity and developmental neurotoxicity in offspring

No adverse effects on postnatal survival and body weight gain until weaning, indicated by unchanged viability and lactation indices, on anogenital distance/index, presence of nipples/areolas, neuronal development and function up to weaning as well as on clinical and gross necropsy examinations was observed in F1 offspring (SI, Additional file 3: Table S16). Devolopmental neurotoxicity examinations comprised brain weight, length and width measurements as well as neuropathological examination by light microscopy and morphometry. Single statistical significance was seen in morphometry, mostly unilateral. Given that the brain weights, length and width measurement and brain histopathology did not show any deviations from the control and no increased zinc content was detected in the brain, it is unlikely that the test items were the cause of this finding. The measurement of thyroid hormones revealed no effect caused by all test items, neither in F0 parental animals nor in F1 offspring. Thus, the NOEC of developmental toxicity and developmental neurotoxicity in the F1 progeny is 10 mg/m^3^ for cZnO and uZnO.

### Toxicity in pups on PND 22

After inhalation exposure of pups (PND 4–22) to the highest test concentration of uZnO, cZnO and µZnO (10 mg/m^3^) as well as ZnSO_4_ (22 mg/m^3^) no systemic toxicity was observed based on clinical examination, gross necropsy, and histological examination of non-respiratory organs. Local toxicity was however observed in lungs and nasal cavities: Minimal cellular debris and neutrophil infiltration in the lungs as well as minimal to moderate degeneration/regeneration of the olfactory epithelium in the nasal cavity was found after the exposure to the highest test concentrations (Additional file 3: Tables S17–S18). The NOAEC for local toxicity in pups is 2 mg/m^3^ for uZnO; no NOAEC could be determined for cZnO.

### Organ burden

**Fig. 7:**
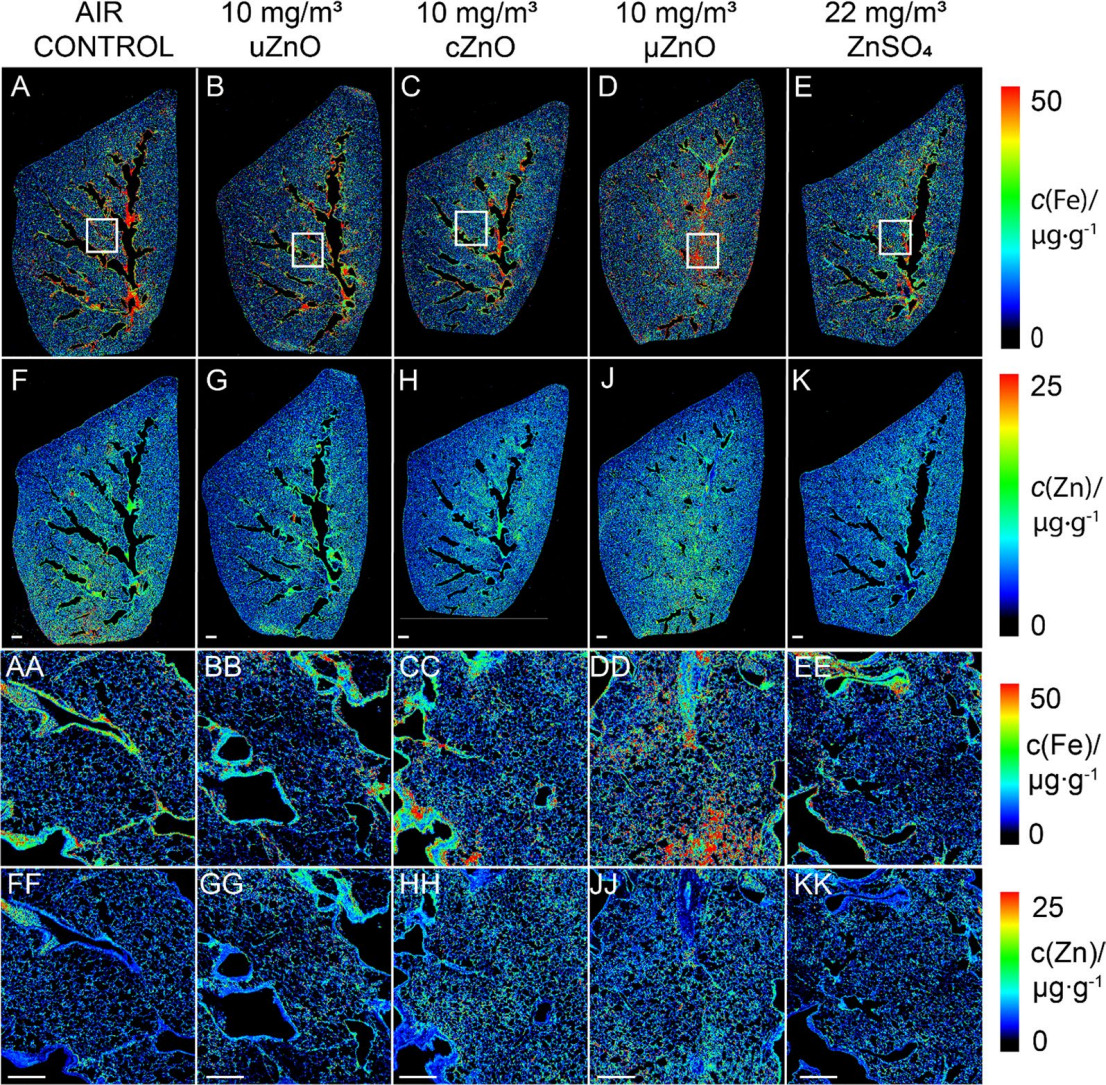
LA-ICP-MS analysis of lungs of control and high concentration group animals after 90-day inhalation exposure. Scale bar **A**–**K** 1 mm, **AA**–**KK** 500 µm. The panels of the first column (**A**, **F**, **AA** and **FF**) were from one lung lobe of a control animal, analyzed for Fe (panel **A**, overview, **AA** high resolution analysis of the indicated region in **A**); analyzed for Zn (panel **F**, overview, **FF** represented the high resolution analysis), The panels of the second column (**B**, **G**, **BB** and **GG**) were from one lung lobe of an animal exposed to 10 mg/m^3^ uZnO, those of the third column from one animal exposed to 10 mg/m^3^ cZnO, those of the fourth column from an animal exposed to 10 mg/m^3^ µZnO, and those of the fifth column from an animal exposed to 22 mg/m^3^ ZnSO_4_

Lungs, liver, heart, brain, and olfactory bulb were examined by ICP-OES to determine the Zn load in each organ resulting from exposure to the test substances. High endogenous Zn levels in the respective organs, except for the olfactory bulb, were detected in control animals. Slightly increased Zn concentrations (less than twofold compared to control animals) above the high background were observed in lungs of animals exposed to high test concentration levels (10 mg/m^3^ ZnO materials, 22 mg/m^3^ ZnSO_4_) of all test substances (see Table 4). Conversely, statistically lower Zn concentrations were found in liver and heart of female animals exposed to cZnO. Spatially dissolved analysis of Zn was performed in lung slices using laser ablation and inductively coupled plasma mass spectrometry analysis (LA-ICP-MS). The Fe distribution was determined mainly to visualize the tissue structure. Moreover, it may be used to take possible inflammatory reactions into account. Fe is an important cofactor for many proteins [67] involved in e.g., oxygen transport or energy storage. In the presented lung tissue (Fig. 7, panels A–E, AA–EE), Fe exhibits an inhomogeneous distribution across the tissue sample in both control and treated animals. However, no histologically relevant structures correlated with the obtained elemental distribution. As previously shown by ICP-OES, the control animal showed already very high background Zn content, that did not seem to be evenly distributed. On the overview image of the LA-ICP-MS analysis (Fig. 7, panels F–K), a comparable pattern of Zn was observed among the examined animals, there were no difference between the animals exposed to ZnO nanoforms or µZnO, or ZnSO_4_. On the high-resolution analysis (Fig. 7, panels FF–KK) there were small regions with slightly higher Zn content, which seemed to be diffuse rather than focused as hot spot. These analyses indicate that although Zn content increased in exposed animals, as already shown in ICP-OES analysis, it was not very likely to be particular. Considering the sub-chronic exposure period of 90 days, this shows the rapid dissolution of the ZnO particles at the portal of entry. In all other examined groups, no changes in organ burdens were detected compared to unexposed animals. ICP-OES analysis data of the organ burden are summarized in the SI, Additional file 3: Table S19.

**Table 4:** ICP-OES analysis of Zn content in lungs of parental Wistar rats after 90-day inhalation exposure (all values as µg)

	Control	uZnO	cZnO	µZnO	ZnSO_4_
Conc. (mg/m^³^)	0	10	10	10	22
Male	20.0 ± 0.8	36.3 ± 1.7**	32.7 ± 2.9*	41.0 ± 2.2**	29.0 ± 2.9*
Female	18.3 ± 0.5	25.7 ± 0.9**	25.7 ± 2.5*	32.0 ± 5.4	26.3 ± 0.5**

Statistical analysis: Welch test: *p < 0.05; **p < 0.01, values are given as mean ± SD, n = 3

### Genotoxicity

After 14-day inhalation exposure to 8 mg/m^3^ uZnO, cZnO, µZnO, and an equimolar concentration of 18 mg/m^3^ ZnSO_4_, changes in BALF were comparable to those of the 90-day study which is displayed in Table 5 (mean x-fold changes are shown in Additional file 3, Table S9).

**Table 5:** Lavage parameters after 14-day inhalation exposure for the in vivo comet assay

	Male animals (n = 5)								
	Control	uZnO			cZnO			µZnO	ZnSO_4_
Concentration (mg/m^3^)	0	0.5	2	8	0.5	2	8	8	18
TCN (cn/µL)	38.82	34.39	41.19	157.78**	39.12	29.87	115.05*	337.80**	106.98**
LY (cn/µL)	0.44	0.46	1.01*	5.48**	0.31	0.36	3.13*3	9.84**	3.81**
MPH (cn/µL)	37.66	32.44	31.43	18.08	36.61	27.54	19.04	40.64	16.56
PMN (cn/µL)	0.41	1.07*	8.42*	131.88**	2.04**	1.83**	91.77**	284.69**	85.57**
Relative LY (%)	1.30	1.25	2.45	3.35	0.85	1.25	2.60	3.75**	3.55*
Relative MPH (%)	96.85	94.50	75.35**	11.30**	93.15	91.95*	19.10*	11.65**	16.35**
Relative PMN (%)	1.05	3.15**	21.35**	83.85**	5.60**	6.30**	77.20**	83.65**	79.35**
Total protein (mg/L)	35	35	45	179	114	36	82**	267**	84**
LDH (µkat/L)	0.29	0.30	0.46*	2.22*	0.42	0.70	1.32**	3.83**	1.32**
ALP (µkat/L)	0.49	0.76*	1.10**	7.50**	0.66	0.80**	2.06**	9.35**	2.10**
NAG (nkat/L)	39	39	45	77**	50	40	57	94**	57
GGT (nkat/L)	28	32	66**	100**	45*	45*	77*	117**	109**

Statistical analysis: Wilcoxon test (one-sided) *p < 0.05; **p < 0.01, values are given as mean

Results of the comet assay as well as LMW DNA diffusion assay are summarized in Table 6. The comet assay did not show genotoxic effects after inhalation of any of the test substances. Relevant cytotoxicity in the indicated tissue was ruled out by the negative results of the LMW DNA diffusion assay.

**Table 6:** Results of comet assay and LMW DNA diffusion assay after 14-day inhalation exposure

Test group	Concentration	% DNA tail intensity				% Low molecular weight DNA diffusion			
	(mg/m^3^)	Nasal epithelium	Lung	Liver	Bone marrow	Nasal epithelium	Lung	Liver	Bone marrow
Air control	0	10.7 ± 2.89	5.6 ± 1.50	2.8 ± 1.00	2.9 ± 0.66	30.0 ± 9.19	8.6 ± 1.52	12.6 ± 5.37	14.8 ± 2.17
uZnO	0.5	9.3 ± 2.64	6.9 ± 4.15	3.2 ± 0.53	2.9 ± 0.52	26.8 ± 6.76	11.6 ± 3.29	15.2 ± 6.02	13.6 ± 2.07
	2	9.0 ± 2.86	4.9 ± 1.19	2.7 ± 0.38	2.8 ± 0.76	33.8 ± 8.98	11.4 ± 2.51	13.2 ± 3.35	14.0 ± 3.54
	8	8.3 ± 1.69	5.4 ± 2.89	3.8 ± 1.19	2.7 ± 0.42	35.8 ± 16.40	10.6 ± 1.52	13.4 ± 6.95	13.8 ± 5.02
cZnO	0.5	10.8 ± 5.05	5.3 ± 2.67	3.1 ± 0.80	2.9 ± 0.68	31.6 ± 8.62	11.8 ± 3.56	12.6 ± 6.80	12.0 ± 3.54
	2	9.9 ± 4.03	4.5 ± 1.34	3.6 ± 0.73	2.9 ± 0.67	36.8 ± 15.67	8.4 ± 3.36	10.0 ± 4.30	16.8 ± 5.89
	8	8.8 ± 3.91	4.2 ± 1.60	3.1 ± 0.83	2.6 ± 0.75	34.6 ± 14.24	10.4 ± 2.61	11.0 ± 3.74	15.2 ± 5.40
µZnO	8	7.8 ± 2.13	4.0 ± 1.60	3.2 ± 1.11	2.2 ± 0.54	29.0 ± 11.05	12.6 ± 4.22*	11.8 ± 4.09	15.6 ± 5.98
ZnSO_4_	18	7.6 ± 1.28*	4.1 ± 1.51	3.1 ± 1.26	2.5 ± 0.37	33.0 ± 8.80	12.0 ± 5.92	10.6 ± 7.64	14.6 ± 6.66
Positive control (EMS)	300 mg/kg b.w	32.3 ± 4.12**	52.4 ± 7.8**	30.7 ± 3.07**	30.6 ± 5.53**	39.8 ± 4.49**	26.8 ± 13.37*	38.2 ± 7.05**	34.6 ± 6.73**

Results are presented as the mean of medians ± SD, (n = 5)

Statistical analysis: Dunnett test (two-sided) for each test substance/t-test (one-sided) for positive control, *p < 0.05; **p < 0.01

### Summary of the results

This study is a comprehensive study to investigate the similarities between two ZnO nanoforms. To facilitate the comparison and present the overall results, a table with the relevant NOAECs for the examined parameters is provided (Table 7.).

**Table 7:** Summary of the results

Test substance	Target concen-trations (mg/m³)	Local effects	Systemic toxicity		Fertility and reproductive performance	Developmental toxicity and developmental neurotoxicity in offspring	Local toxicity in pups on PND 22	Systemic toxicity in pups on PND 22 (based on clinical examination)
		BAL, histopathology of the respiratory tract	Hematology	Clinical chemistry, thyroid hormones, gross necropsy, histology of remote organs according to OECD TG 413	Mating index, litter size and body weight at birth, pup viability, sex ratio of the pups, histology of the reproductive organs in the F0 rats	Postnatal survival, body weight gain, viability, lactation indices, anogenital distance/index, presence of nipples/areolas, thyroid hormones, neuronal development and function, clinical and gross necropsy examinations, brain weight and measurements, neuropathological examination by light microscopy and morphometry	Histology of the respiratory tract	Clinical examination, gross necropsy, histological examination of non-respiratory organs
**uZnO **	0.5, 2, 10	NOAEC ♂ = 0.5 mg/m³ ♀ = n.d. (minimal finding in the nasal cavity in one ♀)	NOAEC (♂♀) ≥ 10 mg/m³ (htc)		NOEC (♂♀) ≥10 mg/m³ (htc)		NOAEC (♂♀) = 2 mg/m³ (findings in the nasal cavity and lungs)	NOAEC (♂♀) ≥ 10 mg/m³ (htc)
**cZnO **	0.5, 2, 10	NOAEC (♂♀) = 0.5 mg/m³	NOAEC (♂♀) ≥ 10 mg/m³ (htc)*		NOEC (♂♀) ≥ 10 mg/m³ (htc)		NOAEC (♂♀) n.d. (findings in the nasal cavity at 0.5 mg/m³)	NOAEC (♂♀) ≥ 10 mg/m³ (htc)
**µZnO**	10	n.a.	NOAEC (♂♀) ≥ 10 mg/m³		NOEC (♂♀) ≥ 10 mg/m³		n.a.	NOAEC (♂♀) ≥ 10 mg/m³
**ZnSO**_4_	22	n.a.	NOAEC (♂♀) ≥ 22 mg/m³		NOEC (♂♀) ≥ 22 mg/m³		n.a.	NOAEC (♂♀) ≥ 22 mg/m³

n.a. not applicable, because only one concentration was tested, and adverse findings were observed

n.d. not determine

htc: highest tested concentration

NOEC: no observed effect concentration

NOAEC: no observed adverse effect concentration

WBC: white blood cells, NG: neutrophilic granulocytes, LY: lymphocytes

* ♂ = 2 mg/m^3^ considering increased WBC, NG, LY in blood, which were considered secondary to the local effects

## Discussion

The goal of this research was to produce reliable data to justify the grouping of different ZnO nanoforms into a set of nanoforms. Previous studies have found evidence for a rapid dissolution of ZnO, releasing Zn ions which makes these likely to be the primary cause of ZnO-induced toxicity [60, 68–72]. The dissolution of ZnO depends on pH, particle size and surface coatings [71, 73, 74]. Given the variety of ZnO nanoforms and the associated effort to sufficiently investigate human health hazards, a grouping approach could support reduced testing and still ensure a reliable risk assessment. To justify grouping a set of similar ZnO nanoforms, we investigated uZnO and cZnO—manufactured from identical starting material—under consideration of size and solubility, including µZnO and soluble ZnSO_4_ as reference substances. The TEM size analysis confirmed that the size distributions of the two ZnO nanoforms were identical, as expected (Fig. 1B). The CFS dissolution analysis confirmed that the coating delayed the dissolution, but that both nanoforms dissolved within few days in lysosomal simulant fluid (Fig. 1C).

### General considerations

The generation of an appropriate test atmosphere is one of the most important technical issues of an inhalation study. In the past, considerable efforts were dedicated to generating atmospheres that contain primary particles. This was based on the belief that the toxicokinetic and toxicodynamic properties of nanoparticles differ significantly from those of larger particles. In some cases, coating or dispersion were even used to prevent agglomeration of nanoparticles. We have chosen commercially available test materials to generate aerosols applying the dry powder generation method. This method does not alter the surface of the particles. Although the particles agglomerated, it is representative for occupational exposure; the agglomerates were highly respirable.

The range of exposure concentrations should cover a range from the Maximum Tolerated Concentration (MTC) down to concentrations of actual human exposures which are between 0.04 and 5 mg/m^3^ [13]. The MTC is defined as high concentration used in repeated exposure toxicity testing that is expected to produce a clear level of toxicity but not cause lethality or persistent signs that might lead to lethality or prevent a meaningful evaluation of the results when administered for the duration of the test period [75]. However, the MTC concept presented is adopted from oral studies that were established decades ago [76]. Compared to the gastrointestinal epithelium in oral studies, the respiratory tract as portal of entry is often more vulnerable. This limits the selection of the highest concentrations due to local toxicity in the respiratory tract. As a result, the internal dose at the highest concentration of inhalation studies can be lower than the internal dose of the highest external dose in oral studies [77]. Since animals of the range-finding study being exposed to a target concentration of 12 mg/m^3^ ZnO (measured concentration: 10.9 mg/m^3^ uZnO, 10.8 mg/m^3^ cZnO) did already suffer from severe local effects, a concentration of 10 mg/m^3^ was selected as the highest concentration in the sub-chronic study.

Nose-only exposure is recommended by OECD guidelines as the preferred exposure mode. Whole-body exposure is, however, less stressful, and suitable for reproductive studies including exposure of pups. With whole-body inhalation exposure, cross-exposure via dermal and oral route (due to grooming) occurs. For ZnO, it is not expected to cause toxicity through dermal absorption [6, 23, 78, 79]. Additionally, a repeated dose oral study in rats with ZnO nanoparticles revealed a no-effect level of 31 mg/kg body weight [20]. While the oral uptake during whole-body inhalation exposure has not been specifically assessed, it is anticipated to be significantly lower than the doses used in the oral study. It is important to consider oral uptake as it contributes to the systemic availability of Zn, ensuring that the potential systemic toxicity of the different forms of ZnO is not underestimated. However, it is worth noting that the whole-body exposure mode does not bias the assessment of local toxicity.

### General, systemic toxicity

Flu-like symptoms have been reported in welders exposed to metal oxides, such as ZnO fumes during welding, thermal cutting or melting [80]. While the regular exceedance of workplace limits for ZnO has been recently discussed [13], further studies showed that already low doses that do not induce metal fume fever lead to inflammation and systemic, acute phase response—determined by serum amyloid A (SAA) and C-reactive protein (CRP)—in human [11] and mice [73]. Vogel et al. consider this effect as adverse and derived a human exposure level based on these parameters [81]. Since these markers are not part of the current OECD test guidelines, we did not include SAA and CRP in the current study. However, we consider the acute inflammation by measured BALF parameters such as PMNs, which were elevated at 2 mg/m^3^ and higher. This approach is supported by a study of Gutierrez and colleagues [82], which demonstrated that SAA and PMNs in BALF provide similar levels of sensitivity in detecting inflammation in mice after intratracheal instillation of metal oxides. Thus, the setting of NOAEC based on BALF and histopathology does not underestimate the toxicity of ZnO nanoforms in our study.

Here, the systemic toxicity of ZnO was assessed using various methods, including clinical observation, body weight measurements, food consumption monitoring, blood analysis (hematology, clinical chemistry, T4 and TSH levels), and histological examinations of 47 different organs and tissues as per OECD TG 413. No signs of toxicity were observed during clinical examination, except for a slight reduction in body weight gain in animals exposed to ZnSO_4_. The observed salivation was considered as being caused by local irritation of the substance in the respiratory tract. The effect on body weight was deemed non-adverse since the decreased body weight gain of uZnO, cZnO, and µZnO exposed animals was transient, and the mean body weights were not different to controls at the end of exposure. The body weight change in ZnSO_4_ exposed animals was less than 10%, which was considered not adverse. In clinical pathology, only males exposed to 10 mg/m^3^ cZnO exhibited slight increases in total white blood cell count, as well as absolute neutrophil and lymphocyte counts, indicating a marginal acute phase reaction, which was considered secondary to the local irritation. No significant changes in clinical pathology parameters were observed at the lower cZnO concentrations or at any concentration of the other test substances. Similarly, no changes were observed in T4 and TSH levels in parental males. These findings support the results of a 90-day inhalation study conducted by Fraunhofer ITEM [15] on cZnO (NM-111) and µZnO (NM-113), as well as a 28-day study on µZnO performed at BASF SE [19]. Chuang et al. [83] found that SD rats exposed to 1.1 or 4.9 mg/m^3^ uZnO (50 nm) for 14 days experienced mild to moderate inflammatory cell infiltration and necrosis in cardiac tissue. These effects progressed to focal fibrosis after 7 days and cardiac tissue necrosis after 30 days post-exposure. Furthermore, Wang et al. [84] reported significant liver damage in rats exposed to intranasal instillation of nano ZnO (20 nm) for three consecutive days, with observed inflammation, interstitial hyperemia, fatty degeneration around the central vein, and expanded hepatocyte necrosis. Of note, Chuang's study did not report any changes in the liver, while Wang’s study did not document any lesions in cardiac tissue. In our present study, we investigated effects at higher concentrations and after longer exposure duration than the previous studies and did not observe any of these effects in either the heart or liver.

Further evidence on systemic toxicity is provided by oral studies. The oral 90-day studies, conducted by Kim et al. [20] and Park et al. [21, 22], did not demonstrate any cardiac or hepatic damage. However, these studies did reveal lesions in the stomach, pancreas, and eyes at doses of 125 mg/kg b.w./day and higher. Kim et al. also identified the prostate gland as a target organ and a NOAEC of 31.25 mg/kg b.w./day was reported. The maximum deposited amount of ZnO at the high concentration of 10 mg/m^3^ in our inhalation study would be approximately 2.4 mg/kg b.w./day (assuming a 100% deposition rate and a minute ventilation volume of 0.2 L per minute). Hence the studies of Chuang et al. [83] and Wang et al. [84] were not reproducible by other researchers, including the study presented here, which used even higher concentrations, thus higher daily internal doses of ZnO. Absence of effects, as seen in the oral studies, is plausible. However, we do not want to speculate as to why some studies come to different conclusions but would like to highlight that our study was performed under the current GLP and OECD guidelines in a DIN EN ISO/IEC 17020 and 17025 accredited facility.

### Local effects at the respiratory tract in adult animals

Larynx, lungs, mediastinal lymph nodes, and nasal cavities were the target organs of local toxicity. Except for the mediastinal lymph nodes, these tissues line the airways which are the sites of first contact and potentially the portals of entry by inhalation exposure. Particles deposit due to their aerodynamic properties, which depend on factors such as particle size, density, and shape [85]. The observed local effects are similar across the two ZnO nanoforms but also with µZnO. Only minor differences in incidence and severity, mainly in the nasal cavity and larynx, were observed after the inhalation of equimolar concentrations of the soluble salt ZnSO_4_.

In the nasal cavity, ciliated columnar and olfactory epithelia are the most fragile types of epithelia of the respiratory tract and thus the most susceptible to damage from inhaled substances [86, 87]. Histopathological examinations of the nasal cavity revealed the loss of olfactory epithelium with occasional regeneration in male and female animals exposed to high concentrations of all test substances. These lesions were still observed in single animals exposed to mid concentration (2 mg/m^3^) of cZnO as well as in one female animal exposed to low concentration (0.5 mg/m^3^) of uZnO. The most severe effects were seen in animals exposed to ZnSO_4_. However, no effects were observed in animals exposed to µZnO. Since previous studies have already demonstrated olfactory damage after intranasal application of ZnSO_4_, we suggest that the local effect is due to Zn ions rather than particles [88, 89].

**Fig. 8:**
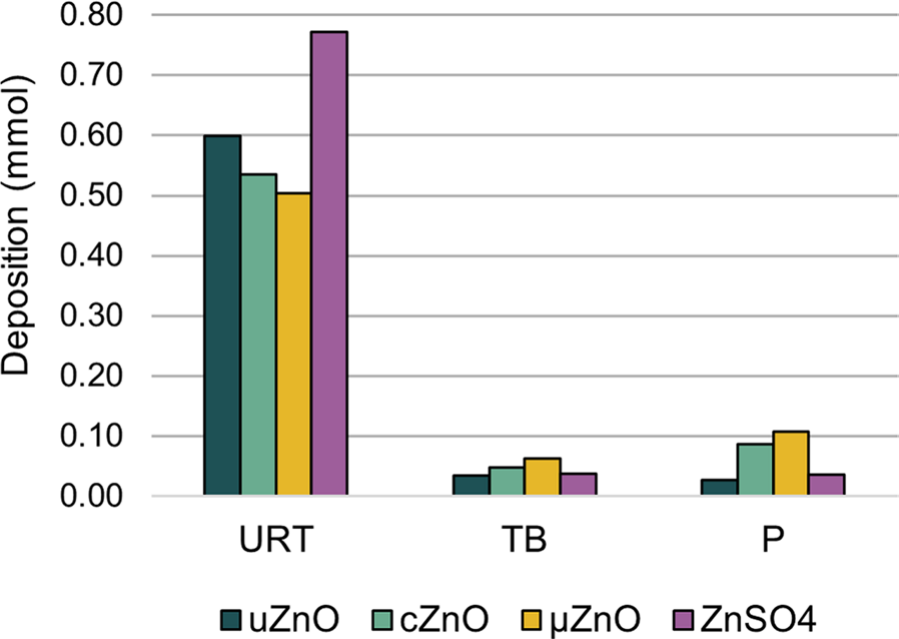
Calculated amount of deposited uZnO, cZnO, µZnO, and ZnSO_4_ in different regions of the respiratory tract. Deposition after 90-day inhalation exposure at high concentration (10 mg/m^3^ ZnO materials and 22 mg/m^3^ ZnSO_4_). URT: upper respiratory tract (nasal cavity, larynx, and trachea), TB: tracheobronchial region, P: pulmonary region

Inhalation of cZnO, uZnO and µZnO caused epithelial alterations of the upper airways which were considered non-adverse, whereas exposure to ZnSO_4_, caused squamous metaplasia at the base of epiglottis and inflammatory cell infiltrates. Moreover, one female animal exhibited erosion/ulceration in the larynx. These differences between particulate ZnO and dissolved Zn ions are the result of the different deposition patterns and likely due to differences in Zn ion release in the nasal cavity. Multiple-path particle dosimetry (MPPD) modeling (Fig. 8) showed that the highest deposition of particles in the head region (including the nasal cavity, larynx, and trachea) was found for ZnSO_4_, while the lowest deposition was observed for µZnO.

In the lungs, the lavage parameters, including PMN, total protein, and LDH, showed an increase in a concentration-dependent manner in animals exposed to both ZnO nanoforms, µZnO and ZnSO_4_. These findings were consistent with the higher lung weights observed at higher concentrations and the histological results, which revealed the presence of foamy macrophages, cellular debris, multi-focal inflammatory cell infiltration, and type II cell hyperplasia at varying levels of occurrence and severity. Foamy macrophages have been observed in several inflammatory conditions as part of the early phase response to foreign matter such as pathogens and particles [90–92]. While alveolar macrophages play a crucial role in the removal of foreign matter from the peripheral lung, excessive exposure to particles [93] or irritant toxicants [94] can lead to an impairment of their efficacy. The increased number of foamy macrophages in the alveoli led to the recruitment of PMNs which further contributed to the inflammatory response by releasing ALP and recruiting monocytes, which differentiate to macrophages in the lung tissue to eliminate cellular debris [95]. As part of the pulmonary clearance, macrophages transported phagocytosed particles (test substance as well as cell debris) to draining, lung-associated lymph nodes (mediastinal and tracheobronchial) which activation was emphasized by lympho-reticular hyperplasia. Hyperplasia of type II pneumocytes indicates the regeneration and replacement of alveolar epithelium after lung injury. Elevated levels of total protein in BALF are a result of an increase in inflammatory proteins but moreover of albumin infiltrating the alveolar space due to vascular leakage in capillaries. Cell death is underlined by the increased LDH levels in lung lavage which is released from cytoplasm after plasma membrane damage [96, 97]. The histological findings were qualitatively comparable with the studies by Fraunhofer ITEM and BASF SE [14, 15, 19]. Overall, the severity of the observed findings in animals exposed to ZnSO_4_ appears to be less than in animals exposed to equivalent molar concentrations of any form of ZnO. This difference can be attributed to the particle size present in the atmosphere, as indicated by the MPPD modeling results discussed earlier for the upper respiratory tract (Fig. 8). It is worth noting that the local effects discussed in this study were not specific for particles, they can be also observed after exposure to irritant gaseous substances such as chlorine, ozone, or nitrogen oxide species [92, 98–100].

All the effects observed in this study were indicative of acute inflammatory responses. However, it is worth mentioning that these effects were found to be reversible within an 8-week period following the exposure. This rapid reversibility can likely be attributed to the rapid dissolution of the ZnO forms, which is also evident for the soluble salt ZnSO_4_, which was tested equimolarly to the high concentration of any forms of ZnO. Similar effects and rapid reversibility were also reported in studies by Fraunhofer ITEM and BASF SE [14–16, 19]. These findings fit into the previously described 5-phase concept of inflammation upon inhalation exposure to particles [101].

Poland et al. [101] stated that particle effects can be discussed in a scientific or regulatory context. While scientifically any deviation from control values may be considered an effect, a regulatory perspective should focus on the prevention of effects in humans, thus the definition of adversity should follow recommendations of ECHA [102] and SCOEL [103, 104]. Following the analysis of Poland et al. [101], several effects which were observed during the exposure fully regressed and are considered adaptive, showing reparative mechanisms.

### Local effects at the respiratory tract in F1 progeny

In offspring, findings were limited to lungs and nasal cavities with much lower severity and incidence than in parental animals, which can be explained by the shorter duration of exposure in pups (17 days) as in the F0 generation (90 days). Comparing the toxicity of uZnO and cZnO in pups of PND 22, the overall finding in lungs were comparable at the high concentration of all test substances, regardless of particle size and solubility. While lesions in nasal cavity were seen after exposure to cZnO, µZnO as well as soluble ZnSO_4_ and was still observed at the low concentration of 0.5 mg/m^3^ cZnO, no nasal damage was revealed in pups exposed to uZnO.

### Fertility, development, and neurotoxicity

Several in vivo studies reported adverse reproductive effects of ZnO nanoforms by other routes (oral and injection) than inhalation, such as impaired spermatogenesis and testes lesions [42–45], changes in the weight of reproductive organs [42, 46] and affected testosterone levels [44, 48, 49]. Dianová et al. [47] recently reviewed the reproductive toxicity of metal nanoparticles, reporting inflammation of female reproductive organs. In contrast, other studies suggested a protective effect against Doxorubicin-induced testicular toxicity in rats exposed to 3 mg/kg b.w./day nano ZnO for 8 weeks [50] or improvement of male fertility in rabbits after 5-week oral administration of 100 mg/kg b.w./day nano ZnO [51]. Concerns regarding particle-driven developmental toxicity were raised by another in vivo experiment testing nanosized ZnO via oral gavage, reporting increased [105] intra-uterine and perinatal mortality after 2-week oral exposure to 500 mg/kg b.w./day [46]. Furthermore, ZnO nanoparticles were reported in in vivo studies after intranasal and inhalation exposure to be transported into various brain regions along the olfactory nerve [69, 106] and after intraperitoneal exposure causing electrophysiological deficits in the hippocampus affecting spatial learning and memory [107]. Other studies could not observe developmental toxicity in prenatal [28, 29] and postnatal [27] rats after maternal dietary exposure to ZnO.

We investigated the reproductive and developmental toxicity as well as neurotoxicity potential of uZnO and cZnO after 90-day inhalation exposure using a combined study design addressing endpoints referenced in OECD TG 413, 421, 424, and 426. Based on the local effects observed in the 14-day range-finding study, the maximum tolerated concentration for inhalation exposure was tested (10 mg/m^3^ ZnO materials, 22 mg/m^3^ ZnSO_4_), which represents a lower internal dose than internal doses of oral or injection exposures of other studies.

There were no indications from clinical examinations as well as gross and histopathology, that any of the test items adversely affected the fertility or reproductive performance of the F0 parental animals up to and including their administered top exposure levels. Ultimately, adverse effects on the reproductive performance or development could not be reproduced following standardized OECD test guidelines under GLP.

Hence, we suggest that uZnO, cZnO, µZnO particles as well as dissolved Zn ions (derived from ZnSO_4_) do neither interfere with fertility and reproductive performance nor with pre-postnatal development of Wistar rats after inhalation exposure.

### Genotoxicity

The published data on the genotoxic potential of ZnO nanoparticles are inconclusive. Several outcomes of in vitro as well as in vivo studies indicate genotoxicity, accompanied or caused by oxidative stress, oxidative DNA damage, and mitochondrial membrane damage [37, 108–111]. The effects were size- as well as surface-dependent [112–115], while other studies could not find indications of any genotoxicity [36, 116–118].

A comet assay following single intratracheal instillation of cZnO (NM-111) in mice at concentrations of up to 100 µg nano ZnO/mouse detected DNA damage in lung lavage cells immediately after exposure but possibly interfering with cytotoxicity [70]. Whereas another study did not observe dose-dependent genotoxicity in murine BALF cells, lung, and liver after intratracheal instillation of low doses (up to 2 µg/mouse) of uZnO (NM-110) and cZnO (NM-111) in mice [73]. Three oral studies reported conflicting outcomes. One study provided oxidative DNA damage in murine liver tissue after 14-day exposure to 50 and 300 mg/kg b.w. of ZnO nanopowder (30 nm) using an fpg-modified comet assay as well as lipid peroxidation assay [119]. Another study tested 500, 1000, and 2000 mg/kg b.w. in rats (SD), did not find any genotoxicity in liver or stomach after oral gavage for four different ZnO nanoforms, differing in size (20 and 80 nm) and surface charge, neither in the comet assay nor in the micronucleus test [116]. A third study, which administered ZnO nanorods (20 nm) in drinking water, was also negative but missed inclusion of positive controls [117]. Finally, in contrast to cZnO, uZnO dose-dependently induced chromosome aberration and micronuclei in bone marrow and peripheral erythrocytes, respectively, following intraperitoneal injection of mice at concentrations of up to 100 mg/kg b.w. [108]. In the same study, a hormetic dose response was observed in liver cells, resulting only at the lowest test concentration of 25 mg/kg b.w. in a statistically significant mutagenic effect. Fadoju et al. [118] on the other hand could not observe micronuclei induction in bone marrow of mice intraperitoneally exposed to up to 150 mg/kg b.w. nanosized ZnO (< 100 nm).

In the present study, we investigated the genotoxic potential of ZnO particles after inhalation exposure considering particle size and solubility using an in vivo mammalian alkaline comet assay according to OECD TG 489.

The range-finding study already revealed significant degeneration/regeneration of the olfactory epithelium and alterations that were indicative of inflammatory responses in the lung after 14-day exposure to 10.9 and 10.8 mg/m^3^ uZnO and cZnO, respectively, which could impair the comet assay. Thus, the top exposure concentration was slightly reduced to 8 mg/m^3^. At this level in BALF, several parameters were increased in a concentration related manner in animals exposed to uZnO and cZnO, indicating the tissues were sufficiently exposed. These findings were comparable with those observed previously in the 14-day range-finding study with these two test materials. Based on the similarity of the observations made in BALF in the 14-day comet assay study and the range-finding study, it can be assumed that the histopathological alterations induced in the comet assay study would also be similar to those observed in the range-finding experiment. Therefore, these data confirm that the study was performed at the highest possible dose avoiding confounding effects in the comet assay.

uZnO and cZnO nanoforms, µZnO particles as well as Zn ions (derived from readily dissolving ZnSO_4_) did not show genotoxic potential in nasal epithelium, lung, liver, and bone marrow of rats after 14-day inhalation exposure at the tested concentrations (up to 8 mg/m^3^ ZnO equivalents). These findings are supported by the negative chromosomal aberration testing in mammalian cells (V79) performed by Fraunhofer ITEM [32]. With regard to particle size and physicochemical properties of ZnO subsets, no pronounced difference was found between uncoated and coated nano ZnO as well as between nano and micron-sized ZnO particles.

### Dissolution, no particles can be detected in target tissues

After termination of the inhalation exposure, lung, liver, heart, and brain were analyzed by ICP-OES. To assess the total amount of Zn content, target organs (lung, liver, heart, and brain) were completely digested and analyzed by ICP-OES. This method is a highly standardized and highly sensitive method to analyze metal content in tissues. As expected, the data showed a very high biological background level of Zn. In the high concentration groups, only a slightly increased Zn concentration was found in lungs. Statistically lower Zn content was found in liver and heart of female animals exposed to 10 mg/m^3^ cZnO, which was considered incidental. In all other examined organs, the Zn level was comparable with the control. The low lung burden despite a 90-day inhalation exposure to high concentration of Zn compounds is a consequence of the high dissolution rate and subsequent rapid clearance. The analysis of the animals with medium and low concentrations was not carried out, as the Zn content was presumably within the range of the control animals. Based on the available data, no increased lung burden was expected after the recovery period. Therefore, no animals of the recovery group were examined for organ burden, thus clearance half-times were not determined.

In addition to conducting ICP-OES analysis, we also performed spatially resolved analysis of Zn in representative animals from the control group and one animal from each of the high concentration groups. The results revealed that control animals already exhibited high background levels of Zn, as indicated by the overview showing Zn content (Fig. 7, panels F–K). Interestingly, in certain regions of both the control and high concentration groups exposed to different forms of ZnO, a slightly uneven distribution of Zn was observed. We attributed this finding to the sample preparation process, specifically the random cutting of the samples, rather than it being indicative of any significant histological differences. Additionally, there was no indication for the presence of Zn as particulates species during the high-resolution analysis (Fig. 7, panels FF–KK). Overall, the data from the LA-ICP-MS analysis were consistent with those obtained from the ICP-OES analysis. A similar conclusion was drawn by the authors who performed the previous 90-day inhalation study with ZnO NM-110 [10].

In the LA-ICP-MS analysis, we also investigated the presence of Fe to visualize the tissue structure. Our imaging analysis suggested that high concentrations of Fe coincided with high concentrations of Zn ions, which could also be seen in control animals. It is well known that the inflammatory response can modify Fe regulation in tissues. Under chronic inflammation conditions a shift of Fe from the circulation to storage sites of the reticuloendothelial system occurs resulting in hypoferremia and hypoferritinemia [120]. In our study, the inflammation response was transient and a correlation between Fe distribution and inflammation response could not be confirmed.

## Conclusions

In conclusion, our study found that uZnO and cZnO nanoforms exhibited similar local effects in the nasal cavity and lungs. The difference observed with uZnO is based on a mild finding of only one animal and is not considered a substantial difference. These local effects were observed to be reversible within the 8-week post-exposure period. Despite the high concentrations of ZnO (10 mg/m^3^) and ZnSO_4_ (22 mg/m^3^) used in the study, the lung burden at the end of the exposure period was only twice as high as the biological background level observed in the control group. Interestingly, no particles were detected after exposure to either of the three ZnO particles, using high-resolution LA-ICP-MS. Obviously, neither coating (cZnO) nor larger size (µZnO) prevented these particles from a rapid dissolution in the airways just as uZnO. Even though the abiotic dissolution kinetics of uZnO and cZnO differed significantly, both were confirmed as dissolving within few days also by the abiotic method.

This study did not show any systemic toxicity, reproductive toxicity, developmental toxicity, developmental neurotoxicity, or genotoxicity of the different forms of ZnO and ZnSO_4_. The respective NOAECs were the highest tested concentrations 10 and 22 mg/m^3^ for the different ZnO particles and ZnSO_4_, respectively (equating 8 mg/m^3^ Zn for all test materials).

Finally, based on these findings, we conclude that grouping and applying read-across approaches based on the toxicological and toxicokinetic similarity of the two representatives is justified.

## Supplementary Information

**Additional file 1.** Supplementary information on test item characterization.

**Additional file 2.** Supplementary information on study design and examinations.

**Additional file 3.** Supplementary data to results section.

## Data Availability

The datasets used and analyzed during the current study are available from the corresponding author on reasonable request with permission of International Zinc Association, Brussels, Belgium. The data that support the findings of this study are available from Fraunhofer ITEM but restrictions apply to the availability of these data. Data are however available from the authors upon reasonable request and with permission of Fraunhofer ITEM.

## Abbreviations

ALP: Alkaline phosphatase
BAL(F): Bronchoalveolar lavage (fluid)
BET: Specific surface area
CFS: Continuous flow system
CRP: C-reactive protein
cZnO: Coated zinc oxide nanoform
FOB: Functional observation battery
GD: Gestation day
GGT: γ-Glutamyl transferase
GSD: Geometric standard deviation
H&E: Hematoxylin and eosin staining
ICP-OES: Inductive coupled plasma optical emission spectroscopy
LA-ICP-MS: Laser ablation inductive coupled plasma mass spectrometry
LDH: Lactate dehydrogenase
LMW: Low molecular weight
LOAEC: Lowest observed adverse effect concentration
LY: Lymphocytes
MA: Motor activity
MMAD: Mass median aerodynamic diameter
MPH: Macrophages
MTC: Maximum tolerated concentration
µZnO: Microscale zinc oxide
NAG: N-acetyl-β-glucosaminidase
NO(A)EC: No observed (adverse) effect concentration
nZnO: Nanoscale zinc oxide
OFO: Open field observations
PSD: Particle size distribution
PMN: Polymorphonuclear neutrophils
PND: Post-natal day
SAA: Serum amyloid A
SI: Supplementary information
SMPS: Scanning mobility particle sizer
TEM: Transmission electron microscopy
uZnO: Uncoated zinc oxide nanoform
ZnO: Zinc oxide
