# Supplementary material for: Toxicological inhalation studies in rats to substantiate grouping of zinc oxide nanoforms
Source: Part Fibre Toxicol. 2024 May 17;21:24. doi: 10.1186/s12989-024-00572-y (PMC11100124; doi:10.1186/s12989-024-00572-y)
Supplement: Supplementary file 1 — Additional file 1. Supplementary information on test item characterization. [file 12989_2024_572_MOESM1_ESM.docx]

**Supplementary Information**

**Title: Toxicological inhalation studies in to substantiate grouping of zinc oxide nanoforms**

**Additional File 1**

Additional file 1 includes the following information:

- Additional method description of test item characterization
  - Transmission Electron Microscopy (TEM)
  - Dissolution in Continuous Flow System (CFS)
- Characterization of REACH identified ZnO nanomaterials
- Table S1: Physicochemical properties of all 28 by REACH identified ZnO nanomaterials
- Table S2: Physicochemical properties of the test items

*Transmission Electron Microscopy (TEM***)**

A tip of a spatula of material was dispersed in 5 mL of ethanol. The dispersion was treated for 5 min in an ultrasonic bath. 5 drops of the dispersion were applied between two glass slides in order to create a thin liquid film. A carbon coated TEM grid was dipped onto the film to transfer the particles to the TEM substrate.

The TEM samples were analyzed using a Tecnai Osiris machine (Thermo-Fisher) operated at 200 keV under bright-field (BF) conditions (Spotsize; 100 µm condenser aperture; 20 µm objective aperture). ~10 images per sample were taken using a Gatan 1000XP 2K CCD camera with an acquisition time of 2s at magnifications of 13500x and 26500x.

The particle size was evaluated using the free [ParticleSizer p](https://imagej.net/ParticleSizer)lugin for “imageJ” developed and validated within the EC FP7 project NanoDefine.(1, 2). Automated evaluation is faster, reproducible and limits operator bias to a minimum compared to manual image analysis. We carefully checked that the settings of the Particle Sizer Plugin (1) are robust for the ZnO materials.

*Dissolution in Continuous Flow System (CFS***)**

The flow-through setup was described in detail by several publications as an implementation of a CFS according to ISO TR 19057 (3-5). A ZnO mass of M_0_ = 1 mg was weighed onto a membrane (cellulose triacetate, Sartorius Stedim Biotech GmbH, Goettingen, Germany: 47 mm diameter, 5 kDa pore size), topped by another membrane, and enclosed in flowthrough cells. The flow through cells at 37 ± 0.5 °C were kept upright to ensure that emerging air bubbles can leave the system and do not accumulate within the cell. The flow rate (V) was 48 mL/d; this corresponds to a ratio, SA/V around 0.02 h/cm, which was independently found to lead to correct predictions (6). The PSF medium – previously validated by US-NIOSH for the purpose of particle dissolution (7) and recommended by the ISO 19057 (3) - was employed to simulate the lysosomal compartment at pH 4.5 (4). The programmable sampler drew 10 mL eluates twice per day. These were stabilized by 0.1N HNO_3_ and analyzed by ICP-MS (Perkin Elmer Nexion 2000b).

All ZnO materials dissolved in the lysosomal pH 4.5 conditions with half-times of less than 1 day. The dissolution kinetics were overall compatible with mono-exponential decays, but the hydrophobic coating resulted in a delayed onset of dissolution with a few hours delay against the uncoated nanoform. The fitted halftime can be converted to a dissolution rate k_min_. To complement the evaluation, we also determined from the same data set the highest observed dissolution rate in a single sampling interval, k_max_. Only for perfectly exponential kinetics the two evaluation approaches would match perfectly.

*Characterization of REACH identified ZnO nanomaterials*

An analysis of the dissolution rate, size, and shape of all 28 ZnO nanomaterials identified by REACH was performed in a preliminary study to enable a suitable selection of ZnO nanoforms for the main study. The median of aspect ratios (AR50) was below 3 µm for all ZnO nanoforms, hence all of them were attributed to the spheroidal shape category according to ECHA guidance for nanoforms registration (ECHA, 2019 (updated in 2022)) (8), see Table S1. All samples dissolved in lysosomal pH 4.5 conditions with half-times of less than one day. The dissolution rate and half-time data indicated that there is no significant modulation of dissolution by the shape subcategory. However, for some materials, hydrophobic coating yields to a slow downed dissolution. A limited number of test materials had hydrophilic coatings and were dissolving comparably as fast as uncoated materials. In Table S1, the dissolution rate of all 28 tested ZnO nanoforms is shown, with test material number 22 (uZnO) and 23 (cZnO) being the most interesting pair as these two forms vary in surface modifications and reveal the biggest difference in half-time (factor 4).

Table S1: Physicochemical properties of all 28 by REACH identified ZnO nanomaterials

| ZnO material No. | Coating | Shape (TEM) | Size and shape measured by TEM | | | Dissolution measured by CFS | | |
| --- | --- | --- | --- | --- | --- | --- | --- | --- |
|  |  |  | Diameter D50 | Aspect ratio AR50 | Aspect ratio AR90 | kmax | fitted halftime from exponential decay | kmin calculated from halftime |
|  |  |  | nm | unitless | unitless | ng/cm²/h | days | ng/cm²/h |
| 1 | none | spheroidal | 29.3 | 1.4 | 2.2 | 83.6 | 0.24 | 303 |
| 2 | hydrophobic | spheroidal | 26.1 | 1.4 | 2.2 | 417 | 0.34 | 472 |
| 3 | none | spheroidal | 19.2 | 1.3 | 1.7 | 115 | 0.42 | 207 |
| 4 | none | spheroidal | 22.8 | 1.5 | 2.3 | 315 | 0.26 | 280 |
| 5 | hydrophobic | spheroidal | 18.8 | 1.5 | 2.4 | 562 | 0.28 | 258 |
| 6 | AI doped, uncoated | spheroidal | 21.2 | 1.6 | 2.6 | 341 | 0.23 | 310 |
| 7 | AI doped, hydrophobic | spheroidal | 20.0 | 1.5 | 2.5 | 272 | 0.83 | 87.5 |
| 8 | none | spheroidal | 20.7 | 1.5 | 2.4 | 308 | 0.23 | 318 |
| 9 | hydrophobic | spheroidal | 12.9 | 1.6 | 2.6 | 492 | 0.22 | 335 |
| 10 | hydrophobic | spheroidal | 15.7 | 1.5 | 2.6 | 362 | 0.23 | 315 |
| 11 | none | spheroidal | 24.2 | 1.3 | 1.7 | 78.9 | 0.29 | 286 |
| 12 | none | spheroidal | 21.9 | 1.3 | 1.7 | 153 | 0.22 | 433 |
| 13 | none | spheroidal | 16.5 | 1.3 | 1.7 | 228 | 0.25 | 211 |
| 14 | hydrophilic | spheroidal | 22.7 | 1.3 | 1.7 | 81.5 | 0.25 | 400 |
| 15 | hydrophobic | spheroidal | 16.8 | 1.3 | 1.7 | 61.2 | 0.45 | 125 |
| 16 | none | spheroidal | 96.6 | 1.3 | 1.7 | 304 | 0.24 | 305 |
| 17 | none | spheroidal | 8.9 | 1.3 | 1.7 | 61.1 | 0.21 | 227 |
| 18 | none | spheroidal | 28.0 | 1.4 | 2.0 | 70.5 | 0.23 | 125 |
| 19 | none | spheroidal | 38.4 | 1.5 | 2.2 | 87.9 | 0.23 | 125 |
| 20 | none | spheroidal | 55.0 | 1.3 | 1.6 | 395 | 0.22 | 330 |
| 21 | none | spheroidal | 45.0 | 1.4 | 2.1 | 348 | 0.21 | 350 |
| 22 (uZnO)* | none | spheroidal | 18.2 | 1.3 | 1.7 | 323 | 0.19 | 378 |
| 23 (cZnO)* | hydrophobic | spheroidal | 18.3 | 1.3 | 1.7 | 246 | 0.74 | 97.5 |
| 24 | hydrophobic | spheroidal | 19.8 | 1.3 | 1.7 | 438 | 0.31 | 235 |
| 25 | hydrophilic | spheroidal | 61.6 | 1.2 | 1.6 | 408 | 0.24 | 300 |
| 26 | hydrophilic | spheroidal | 56.9 | 1.4 | 2.1 | 281 | 0.24 | 298 |
| 27 | none | spheroidal | 20.0 | 1.2 | 1.6 | 178 | 0.23 | 504 |
| 28 | hydrophobic | spheroidal | 70.8 | 1.3 | 1.7 | 372 | 0.44 | 165 |

*Material 22 (uZnO) and 23 (cZnO) are the most interesting pair with regards to dissolution rate

Table S2: Physicochemical properties of test items

|  | **Size and shape measured by TEM** | | | **Shape (TEM)** | **Specific Surface Area**  **(BET)** | **Surface modification** | **Dissolution measured by CFS** | | | **Crystallinity/ impurities/**  **purity** |
| --- | --- | --- | --- | --- | --- | --- | --- | --- | --- | --- |
|  | Diameter D50 | Aspect ratio AR50 | Aspect ratio AR90 |  |  |  | k_max_ | Fitted halftime from exponential decay | k_min_ calculated from halftime |  |
|  | nm | unitless | unitless | descriptive | m^2^/g |  |  | days | ng/cm²/h | % |
| **uZnO** | 18.2 | 1.3 | 1.7 | spheroidal | 10 | None | 323 | 0.19 | 378 | 98.2 (ZnO)  Loss on drying 1.0  Loss on ignition 1.5 |
| **cZnO** | 18.3 | 1.3 | 1.7 | spheroidal | 10 | Coated | 246 | 0.74 | 97.5 | 93.8 (ZnO)  Loss on drying 0.3  Loss on ignition 1.8 |
| **µZnO** | *n.d.* | *n.d.* | *n.d.* | *n.d.* | 4.48 | None | *n.d.* | *n.d.* | *n.d.* | 99.8 (ZnO) |
| **ZnSO_4_** | 1080 | *n.d.* | *n.d.* | *n.d.* | *n.d.* | - | *n.d.* | *n.d.* | *n.d.* | 36.2 (Zn) |

n.d. = not determined

**REFERENCES**

1. Verleysen E, Wagner T, Lipinski H-G, Kägi R, Koeber R, Boix-Sanfeliu A, et al. Evaluation of a TEM based Approach for Size Measurement of Particulate (Nano) materials. Materials. 2019;12(14):2274.

2. Mech A, Rauscher H, Rasmussen K, Babick F, Hodoroaba V-D, Ghanem A, et al. The NanoDefine Methods Manual. 2020.

3. ISO/TR19057. Nanotechnologies — Use and application of acellular in vitro tests and methodologies to assess nanomaterial biodurability. ISO/TR. 2017;19057.

4. Zanoni I, Keller J, Sauer UG, Mueller P, Ma-Hock L, Jensen K, et al. Dissolution Rate of Nanomaterials Determined by Ions and Particle Size under Lysosomal Conditions: Contributions to Standardization of Simulant Fluids and Analytical Methods. Chem Res Toxicol. 2022;35(6):963-80.

5. Keller JG, Graham UM, Koltermann-Jülly J, Gelein R, Ma-Hock L, Landsiedel R, et al. Predicting dissolution and transformation of inhaled nanoparticles in the lung using abiotic flow cells: The case of barium sulfate. Scientific Reports. 2020;10(1):458.

6. Keller JG, Peijnenburg W, Werle K, Landsiedel R, Wohlleben W. Understanding Dissolution Rates via Continuous Flow Systems with Physiologically Relevant Metal Ion Saturation in Lysosome. Nanomaterials. 2020;10(2):311.

7. Stefaniak AB, Guilmette RA, Day GA, Hoover MD, Breysse PN, Scripsick RC. Characterization of phagolysosomal simulant fluid for study of beryllium aerosol particle dissolution. Toxicology in Vitro. 2005;19(1):123-34.

8. ECHA. Appendix for nanoforms applicable to the Guidance on Registration and Substance Identification2022 January 2022.
