# Supplementary material for: Toxicological inhalation studies in rats to substantiate grouping of zinc oxide nanoforms
Source: Part Fibre Toxicol. 2024 May 17;21:24. doi: 10.1186/s12989-024-00572-y (PMC11100124; doi:10.1186/s12989-024-00572-y)
Supplement: Supplementary file 2 — Additional file 2. Supplementary information on study design and examinations. [file 12989_2024_572_MOESM2_ESM.docx]

**Supplementary Information**

**Title: Toxicological inhalation studies in rats to substantiate grouping of zinc oxide nanoforms**

**Additional File 2**

Additional file 2 includes the following information:

- Details on housing and diet before, during, and after the exposure period
- Table S3: Detailed study timeline of modified repeated dose 90-day inhalation study
- Table S4: Number of animals selected for examinations
- Details on examination of hematological and clinical parameters:

*Detailed housing and diet information*

Air conditions: Temperature 20 - 24°C, relative humidity 45 - 65%.

15 air changes per hour.

Illumination period: 06.00 a.m. - 06.00 p.m. light,

06.00 p.m. - 06.00 a.m. dark

Type of cage / No. of

animals per cage: From delivery until mating and male animals after mating: Typ 2000P: ca. 2065 cm2 (polysulfone cages) / up to 5 animals
During mating: type III polycarbonate cages, 1 male/1 female per cage

During rearing up to PND 22: type III polycarbonate cages, 1 dam with her litter.

After weaning the females from study day 90 after exposure onward until sacrifice: Typ 2000P: ca. 2065 cm2 (polysulfone cages) / up to 5 animals. Remaining females with litters were maintained in type III cages until weaning.

For Motor Activity Measurement: Type III polycarbonate cages (floor area about 800 cm²) / 1 animal

During Exposure:

Wire cages, type DK III / up to 2 animals

Females from PND 4 until study day 94 (and females without litter from the same time period onwards): perforated polycarbonate cages type II. From study day 95 onward wire cages, type DK III

Enrichment: Wooden gnawing blocks (SAFE® block large) J. Rettenmaier & Söhne GmbH + Co KG, Rosenberg, Germany and Play Tunnel, large (Art. 14153); PLEXX b.v., Elst, The Netherlands

Bedding: Dust-free wooden bedding

Nesting material: Compacted fibers of softwood (Typ SAFE® compact nesting large), J. Rettenmaier & Söhne GmbH + Co KG, Rosenberg, Germany

Type of diet: Mouse and rat maintenance diet “GLP”, 12 mm pellets Granovit AG, Kaiseraugst, Switzerland; *ad libitum* before and after exposure. Food was withdrawn during exposure.

Watering: Drinking water ad libitum before and after exposure
Generally, water was withdrawn during exposure, with the exception for females with litter. They received hydrogel pads (approx. 20 g/cage/day) from PND 14 - 16 onward.

Acclimatization: During the acclimatization period the animals were accustomed to the surroundings of the study and to the diet.

Table S3: Detailed study timeline of modified repeated dose 90-day inhalation study

| **Test Dates** | **Study Section** | **Study Day** |
| --- | --- | --- |
| 24 Nov 2020 | Supply of animals and beginning of acclimatization period | -11 |
| 30 Nov 2020 | Randomization of the animals | -5 |
| 02 Dec 2020 | Acclimatization to the exposure procedure/Beginning of pre-exposure period | -3 |
| 05 Dec 2020 | Beginning of exposure period | 0 |
| 04 Jan 2021 | Beginning of determination of estrous cycle (during premating) | 30 |
| 17 Jan 2021 | First mating of parental animals for the formation of F1 generation pups | 43 |
| From 06 Feb 2021 | Last exposure day F0 females before delivery, gestation day (GD) 19 | from 63 |
| From 07 Feb 2021 | Start of exposure-free period GD 20 | from 64 |
| From 08 Feb 2021 | Parturition of F1 generation pups (postnatal day (PND) 0) | from 65 |
| From 09 Feb 2021 | Anogenital distance of F1 pups (PND 1) | from 66 |
| From 12 Feb 2021 | Culling of F1 litter/blood sampling (PND 4) | from 69 |
| From 12 Feb 2021 | Start of exposure period PND 4 (dams with pups) | from 69 |
| From 22 Feb 2021 | Nipple/areola presence (PND 13)/ nipple/areola presence (either PND 20 or 21)/ OFO; MA pups PND 13 subset IV | from 79 |
| From 26 Feb 2021 | MA pups PND 17 subset IV | from 83 |
| From 02 Mar 2021 | OFO; MA pups PND 21 subset IV/ FOB; MA of male parental animals | from 87 |
| From 03 Mar 2021 | Selection and sacrifice of pups for neuropathology PND 22 subset II/ sacrifice and blood sampling of surplus F1 pups (PND 22) subset I and subset III/ sacrifice of pups for particle detection PND 22 subset V | from 88 |
| From 07 Mar 2021 | End of exposure period of male parental animals, and recovery male and female recovery animals | from 92 |
| From 08 Mar 2021 | Start of the recovery period | from 93 |
| From 08 Mar 2021 | Start of sacrificing, sampling of organs for determination of organ burden | from 93 |
| From 09 Mar 2021 | FOB; MA of female parental animals/ Start of blood sampling, bronchoalveolar lavage and sacrifice of F0 male generation parental animals*, start of pathological and clinical pathological examination | from 94 |
| From 9 Mar 2021 | Start of perfusion fixation** | from 94 |
| From 14 Mar 2021 | End of exposure period (parental females) | from 99 |
| From 15 Mar 2021 | Start of blood sampling, bronchoalveolar lavage and sacrifice of F0 female generation parental animals*, start of pathological and clinical pathological examination | from 100 |
| From 03 May 2021 | Start of sacrificing, sampling of organs for determination of organ burden of recovery group male animals | from 149 |
| From 04 May 2021 | Start of blood sampling, bronchoalveolar lavage and sacrifice of recovery group male animals | from 150 |
| From 05 May 2021 | Start of sacrificing, sampling of organs for determination of organ burden of recovery group female animals | from 151 |
| From 06 May 2021 | Start of blood sampling, bronchoalveolar lavage and sacrifice of recovery group female animals | from 152 |

FOB = functional observational battery, MA = motor activity, OFO = open field observation

* Before blood sampling and necropsy food was withdrawn for about 16-20 hours.
** Before perfusion fixation food was withdrawn for about 16-20 hours.

Table S4: Number of animals selected for examinations

| Animals 14-day study  Range-finding | Number of animals selected | Day of examination | Examination |
| --- | --- | --- | --- |
| Adult | 5 males/group | Day after exposure period | Clinical pathology, histopathology, lavage |
| Animals 14-day study Comet assay | **Number of animals selected** | **Day of examination** | **Examination** |
| Adult | 5 males/group | Day after exposure period | Lavage, pathology, comet assay |
| Animals 90-day study | **Number of animals selected** | **Day of examination** | **Examination** |
| Parental | 10/sex/group | Day after exposure period | Clinical pathology, histopathology, lavage Functional observation battery, motor activity |
| Adult | 5 males and 5 females/control and high concentration group | Day after recovery period  Day -1, 10, 23, 47, 75, 91 | Clinical pathology, histopathology, lavage  Detailed clinical observation |
| Adult | 10/sex/control and high concentration group | Day after exposure period | Ophthalmological changes |
| Parental | 3/sex/group  3/sex/group | Day after exposure period  Day after recovery period | Organ burden |
| Subset 90-day study | **Number of pups selected** | **Day of examination** | **Examination** |
| I | 10/sex/group | PND 22 | Thyroid hormones |
| II | 10/sex/group | PND 22 | Brain weights and neuropathology |
| III | 5/sex/group | PND 22 | Histopathological examination |
| IV | 10/sex/group | PND 13, 21  PND 13, 17, 21 | Open field observation  Motor activity |

*Detailed examination of hematological and clinical parameters*

Hematology (Advia 120, Bayer, Fernwald, Germany) included white and red blood cell count, hemoglobin, hematocrit, mean corpuscular volume, mean corpuscular hemoglobin and hemoglobin concentration, platelets, differential blood cell count as well as the clotting potential via prothrombin time (AMAX destiny plus model; Trinity biotech, Lemgo, Germany). Examined clinicochemical parameters (Cobas c501; Roche, Mannheim, Germany) were alanine and aspartate aminotransferase, alkaline phosphatase, γ-glutamyltransferase, sodium, potassium, chloride, inorganic phosphate, calcium, urea, creatinine, glucose, total bilirubin, total protein, albumin, globulins, triglycerides, cholesterol.
