# Supplementary material for: Toxicological inhalation studies in rats to substantiate grouping of zinc oxide nanoforms
Source: Part Fibre Toxicol. 2024 May 17;21:24. doi: 10.1186/s12989-024-00572-y (PMC11100124; doi:10.1186/s12989-024-00572-y)
Supplement: Supplementary file 3 — Additional file 3. Supplementary data to results section. [file 12989_2024_572_MOESM3_ESM.docx]

**Supplementary Information**

**Title: Toxicological inhalation studies in rats to substantiate grouping of zinc oxide nanoforms**

**Additional file 3**

Figure S1: Increase of absolute neutrophil (PMN) counts in lung lavage fluid after 90-day inhalation exposure at high test concentration (10 mg/m³ ZnO materials, 22 mg/m³ ZnSO_4_)

Table S5: Lung weight and histological findings in the respiratory tract of animals exposed to uncoated and coated nano ZnO for 14 days in the range-finding study.

Table S6: Incidence and severity of main histological findings in lungs, larynx, and nasal cavity of animals exposed to uncoated and coated nano ZnO for 14 days in the range-finding study

Table S7: Lavage parameters after 14-day inhalation exposure to uncoated and coated nano ZnO in the range-finding study

Table S8-S10: Mean changes (x-fold of control) in lavage parameters at the end of exposure

Table S11: Lavage parameters of adult animals after 90-day inhalation exposure

Table S12: Adverse hematological parameters of adult animals after 90-day inhalation exposure

Table S13-S14: Incidence and severity of main histological findings in the respiratory tract of adult animals after 90-day inhalation exposure to nano and microscale ZnO and ZnSO_4_

Table S15: Reproductive performance of adults and litters during inhalation exposure to uncoated and coated nano ZnO, microscale ZnO, and ZnSO_4_

Table S16: Developmental toxicity of F1 progeny after exposure to uncoated and coated nano ZnO, microscale ZnO, and ZnSO_4_

Table S17: Histological findings in the respiratory tract of pups (PND 22) in the modified 90-day repeated dose inhalation study

Table S18: Incidence and severity of main histological findings in the respiratory tract of pups (PND 22) in the modified 90-day repeated dose inhalation study

Table S19: Summary of ICP-OES analysis of zinc content in selected organs of parental male and female Wistar rats 90-day inhalation exposure to uncoated and coated nano ZnO, microscale ZnO, and ZnSO_4_


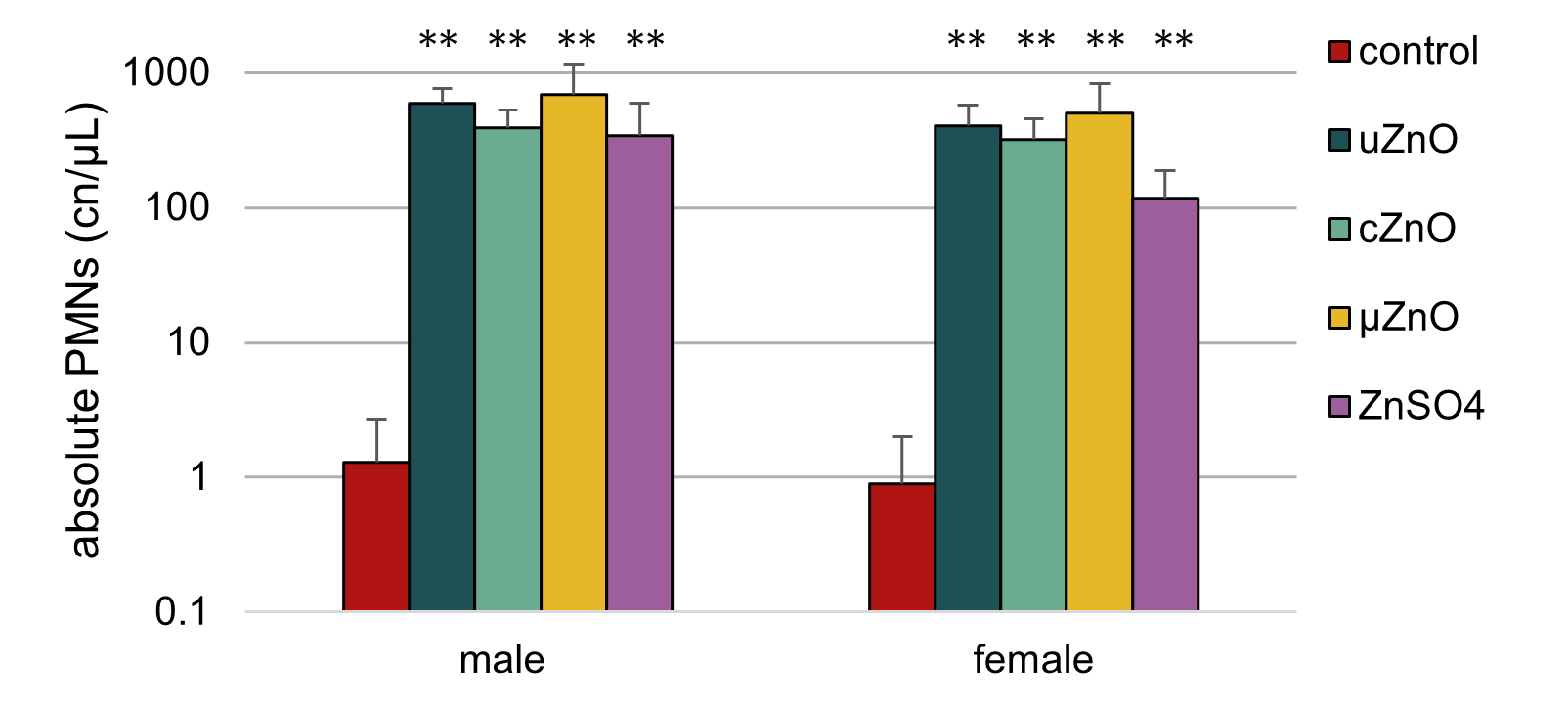


Figure S1: Increase of absolute neutrophil (PMN) counts in BALF after 90-day inhalation exposure at high test concentration (10 mg/m³ ZnO materials, 22 mg/m³ ZnSO_4_). Logarithmic scale. Statistical analysis: dose groups compared to control group, Wilcoxon test (one-sided) *: p < 0.05; **: p < 0.01, values are given as mean with error bars showing standard deviations, n = 10

Table S5: Lung weight and histological findings in the respiratory tract after 14-day inhalation exposure to uZnO and cZnO in the range-finding study

| **Male (n=5)** |  | **control** | **uZnO** | | **cZnO** | |
| --- | --- | --- | --- | --- | --- | --- |
| **Concentration (mg/m³)** | | **0** | **12** | **24** | **12** | **24** |
| **Lung weight changes** | Absolute lung weight | 100% | 113% | 135%** | 114%** | 141%** |
|  | Relative lung weight | 100% | 117% | 138%** | 119%** | 154%** |
| **Histological findings:** | |  |  |  |  |  |
| No. of animals examined | | 5 | 5 | 5 | 5 | 5 |
| **Lungs** | | | | | | |
| Infiltration, granulocytic | | 0 | 1.2 | 2 | 1.4 | 1.6 |
| Histiocytosis, alveolar | | 0 | 2.2 | 3 | 2.2 | 3.4 |
| **Larynx** | | | | | | |
| Epithelial alteration | | 0.2 | 0 | 0.8 | 0.2 | 0.4 |
| **Nasal cavity** | | | | | | |
| Degeneration/regeneration, olfactory epithelium | | 0 | 1.4 | 3 | 2.4 | 3.8 |

Statistical analysis: Fisher’s exact test (one-sided+) was performed based on incidences of the findings.
*: p < 0.05; **: p < 0.01

Table S6: Incidence and severity of main histological findings in lungs, larynx, and nasal cavity after 14-day inhalation exposure to uZnO and cZnO in the range-finding study

| **Male (n=5)** | **control** | **uZnO** | | **cZnO** | |
| --- | --- | --- | --- | --- | --- |
| **Concentration (mg/m³)** | **0** | **12** | **24** | **12** | **24** |
| No. of animals examined | 5 | 5 | 5 | 5 | 5 |
| **Lungs** | | | | | |
| Infiltration, granulocytic | 0 | 5** | 5** | 5** | 5** |
| - Grade 1 |  | 4 |  | 3 | 2 |
| - Grade 2 |  | 1 | 5 | 2 | 3 |
| Histiocytosis, alveolar | 0 | 5 | 5 | 5 | 5 |
| - Grade 1 |  |  |  | 1 |  |
| - Grade 2 |  | 4 |  | 2 |  |
| - Grade 3 |  | 1 | 5 | 2 | 3 |
| - Grade 4 |  |  |  |  | 2 |
| **Larynx** | | | | | |
| Epithelial alteration | 1 | 0 | 4 | 1 | 2 |
| - Grade 1 | 1 |  | 4 | 1 | 2 |
| **Nasal cavity** | | | | | |
| Degeneration/regeneration, olfactory epithelium | 0 | 5** | 5** | 5** | 5** |
| - Grade 1 |  | 3 |  |  |  |
| - Grade 2 |  | 2 |  | 3 |  |
| - Grade 3 |  |  | 5 | 2 | 1 |
| - Grade 4 |  |  |  |  | 4 |

Statistical analysis: Fisher’s exact test (one-sided+), *: p < 0.05; **: p < 0.01

Table S7: Lavage parameters after 14-day inhalation exposure to uZnO and cZnO in the range finding study

| **Male (n=5)** | **control** | **uZnO** | | **cZnO** | |
| --- | --- | --- | --- | --- | --- |
| Concentration (mg/m³) | **0** | **12** | **24** | **12** | **24** |
| TCN (cn/µL) | 68.02 | 505.25* | 607.77** | 327.48** | 588.01** |
| LY (cn/µL) | 2.45 | 11.99** | 18.24** | 9.92* | 18.28** |
| MPH (cn/µL) | 67.15 | 43.66 | 70.80 | 76.97 | 46.31 |
| PMN (cn/µL) | 0.70 | 432.38* | 503.07** | 232.14** | 511.25** |
| Relative LY (%) | 2.94 | 3.00 | 3.10 | 2.90 | 3.10 |
| Relative MPH (%) | 95.88 | 10.25* | 11.90* | 24.40** | 7.55** |
| Relative PMN (%) | 0.94 | 83.17* | 82.45** | 69.90** | 87.40** |
| Total protein (mg/L) | 29 | 365** | 339** | 122** | 425** |
| LDH (µkat/L) | 0.36 | 5.74** | 4.45** | 1.97** | 5.22** |
| ALP (µkat/L) | 0.33 | 6.13** | 6.49** | 2.46** | 8.98** |
| NAG (nkat/L) | 53 | 235* | 127* | 86** | 148** |
| GGT (nkat/L) | 28 | 253** | 111** | 73** | 105** |

Statistical analysis: Wilcoxon test (one-sided) *: p < 0.05; **: p < 0.01

Table S8: Mean changes (x-fold of control) in lavage parameters after 14-day inhalation exposure in the range-finding study

| **Male (n=5)** | **uZnO** | | **cZnO** | |
| --- | --- | --- | --- | --- |
| Concentration (mg/m³) | **12** | **24** | **12** | **24** |
| total protein | 14.4 | 19.6 | 5.4** | 17.5** |
| LDH | 11.0 | 11.2 | 4.6** | 11.7** |
| ALP | 16.6 | 23.5 | 7.0** | 27.4** |
| NAG | 2.7 | 2.7 | 1.7** | 2.8** |
| GGT | 4.6 | 4.1 | 2.9** | 4.3** |
| cell count | 7.4 | 8.9 | 4.8** | 8.6** |
| MPH | 0.1** | 0.1** | 0.3** | 0.1** |
| LY | 1.0 | 1.1 | 1.0 | 1.1 |
| PMN | 88.5** | 87.7** | 74.4** | 93.0** |

Statistical analysis: Wilcoxon test (one-sided) *: p < 0.05; **: p < 0.01

Table S9: Mean changes (x-fold of control) in lavage parameters at the end of exposure in 14-day comet assay study

| **Male (n=5)** | **uZnO** | | | **cZnO** | | | **µZnO** | **ZnSO_4_** |
| --- | --- | --- | --- | --- | --- | --- | --- | --- |
| Concentration (mg/m³) | **0.5** | **2** | **8** | **0.5** | **2** | **8** | **8** | **18** |
| total protein | 1.0 | 1.3 | 5.4 | 3.3 | 1.0 | 2.3 | 7.6 | 2.4 |
| LDH | 1.0 | 1.6 | 7.7 | 1.4 | 1.3 | 4.6 | 13.2 | 4.6 |
| ALP | 1.6 | 2.2 | 15.3 | 1.3 | 1.6 | 4.2 | 19.1 | 4.3 |
| NAG | 1.0 | 1.2 | 2.0 | 1.3 | 1.0 | 1.5 | 2.4 | 1.5 |
| GGT | 1.1 | 2.4 | 3.6 | 1.6 | 1.6 | 2.8 | 4.2 | 3.9 |
| cell count | 0.9 | 1.1 | 4.1** | 1.0 | 0.8 | 3.0** | 8.7** | 2.8** |
| MPH | 1.0 | 0.8 | 0.1** | 1.0 | 0.7 | 0.5 | 0.1 | 0.2** |
| LY | 1.0 | 1.9* | 2.6* | 0.7 | 0.8 | 7.1** | 2.9** | 2.7* |
| PMN | 3.0* | 20.3** | 79.9** | 5.0** | 4.5** | 223.8** | 79.7** | 75.6** |

Statistical analysis: Wilcoxon test (one-sided) *: p < 0.05; **: p < 0.01

Table S10: Mean changes (x-fold of control) in lavage parameters of adult animals after 90-day inhalation exposure

| **Male (n=10)** | **uZnO** | | | **cZnO** | | | **µZnO** | **ZnSO_4_** |
| --- | --- | --- | --- | --- | --- | --- | --- | --- |
| Concentration (mg/m³) | **0.5** | **2** | **10** | **0.5** | **2** | **10** | **10** | **22** |
| total protein | 1.4 | 1.3** | 6.5** | 1.0 | 1.2 | 5.5** | 7.8 | 3.5 |
| LDH | 1.7 | 1.7** | 12.4** | 1.0 | 1.4** | 8.7** | 12.3 | 6.1 |
| ALP | 1.1 | 1.8** | 8.1** | 1.0 | 1.7** | 8.6** | 9.5 | 4.4 |
| NAG | 1.0 | 0.9 | 2.7** | 0.9 | 0.8 | 1.9** | 3.6 | 1.8 |
| GGT | 1.1 | 1.5** | 2.2** | 1.0 | 1.4** | 1.9** | 2.4 | 2.3 |
| cell count | 1.1 | 1.0 | 13.7** | 1.0 | 0.9 | 9.3** | 16.0** | 9.9** |
| MPH | 1.0 | 0.8** | 0.1** | 1.0 | 0.8** | 0.2** | 0.1** | 0.3** |
| LY | 2.2* | 3.3* | 1.3 | 2.3 | 2.3* | 1.5* | 1.4 | 1.7* |
| PMN | 1.8 | 7.2** | 36.2** | 1.4 | 7.6** | 34.7** | 35.0** | 29.4** |
| **Female (n=10)** | **uZnO** | | | **cZnO** | | | **mZnO** | **ZnSO_4_** |
| Concentration (mg/m³) | **0.5** | **2** | **10** | **0.5** | **2** | **10** | **10** | **22** |
| total protein | 1.0 | 1.0 | 4.5** | 0.9 | 1.1 | 4.5** | 7.8** | 2.4** |
| LDH | 0.9 | 1.0 | 5.3** | 1.0 | 1.1 | 5.6** | 8.0** | 3.4** |
| ALP | 1.1 | 1.4** | 7.5** | 1.1 | 1.5** | 7.5** | 10.3** | 3.1** |
| NAG | 1.0 | 0.9 | 1.5** | 0.9 | 1.1 | 1.4** | 1.7** | 1.1** |
| GGT | 1.0 | 1.2 | 2.9** | 1.1 | 1.6** | 2.6** | 3.1** | 2.6** |
| cell count | 0.9 | 0.8 | 7.4** | 0.8 | 0.8 | 5.6** | 8.0** | 2.7** |
| MPH | 1.0 | 0.9** | 0.2** | 1.0 | 1.0 | 0.1** | 0.1** | 0.4** |
| LY | 0.7 | 3.5 | 1.6* | 0.7 | 1.2 | 2.0* | 1.2 | 1.8* |
| PMN | 0.6 | 2.4 | 64.0** | 2.0 | 3.0** | 65.1** | 69.9** | 46.8** |

Statistical analysis: Wilcoxon test (one-sided) *: p < 0.05; **: p < 0.01

Table S11: Lavage parameters of adult animals after 90-day inhalation exposure

|  | **Male animals (n=10)** | | | | | | | | | **Female animals (n=10)** | | | | | | | | |
| --- | --- | --- | --- | --- | --- | --- | --- | --- | --- | --- | --- | --- | --- | --- | --- | --- | --- | --- |
|  | **control** | **uZnO** | | | **cZnO** | | | **µZnO** | **ZnSO_4_** | **control** | **uZnO** | | | **cZnO** | | | **µZnO** | **ZnSO_4_** |
| Concentration (mg/m³) | **0** | **0.5** | **2** | **10** | **0.5** | **2** | **10** | **10** | **22** | **0** | **0.5** | **2** | **10** | **0.5** | **2** | **10** | **10** | **22** |
| TCN (cn/µL) | 50.53 | 55.14 | 49.67 | 693.58** | 48.80 | 47.41 | 468.47** | 806.19** | 502.7** | 70.63 | 65.44 | 57.14 | 520.22** | 59.48 | 54.75 | 398.32** | 563.98** | 191.21** |
| LY (cn/µL) | 1.00 | 2.14* | 2.95* | 14.55** | 2.97 | 2.20 | 12.30* | 17.33** | 16.68** | 2.40 | 1.09 | 12.59 | 23.65** | 1.22 | 1.54 | 21.22** | 14.02** | 8.79** |
| MPH (cn/µL) | 47.56 | 50.36 | 38.00 | 78.81 | 43.59 | 36.00 | 63.61 | 85.49* | 138.06** | 66.57 | 63.51 | 42.74 | 84.07 | 56.18 | 42.41 | 51.32 | 40.82 | 60.43 |
| PMN (cn/µL) | 1.30 | 2.14 | 8.06** | 592.71** | 1.63 | 8.42** | 388.27** | 691.24** | 342.8** | 0.99 | 0.49 | 1.49 | 407.32** | 1.61 | 1.60 | 322.56** | 504.20** | 117.50** |
| Relative LY (%) | 1.80 | 4.00* | 6.00* | 2.38 | 4.20 | 4.18* | 2.65* | 2.55 | 3.02* | 2.60 | 1.80 | 9.14** | 4.08* | 1.78 | 3.14 | 5.20* | 3.02 | 4.75* |
| Relative MPH (%) | 94.52 | 90.65 | 75.80** | 11.52** | 91.35 | 76.40** | 14.75** | 13.82** | 26.82** | 95.40 | 96.42 | 86.61** | 14.82** | 94.67 | 92.39 | 12.65** | 8.62** | 34.45** |
| Relative PMN (%) | 2.35 | 4.22 | 16.90** | 85.02** | 3.18 | 17.80** | 81.55** | 82.15** | 69.08** | 1.25 | 0.78 | 3.06* | 80.05** | 2.56 | 3.72** | 81.38** | 87.32** | 58.52** |
| Total protein (mg/L) | 27 | 37 | 35** | 173** | 26 | 31 | 146** | 208** | 94** | 31 | 32 | 31 | 135** | 28 | 33 | 139** | 243** | 74** |
| LDH (µkat/L) | 0.32 | 0.54 | 0.55** | 3.95** | 0.31 | 0.44** | 2.77** | 3.95** | 1.95** | 0.56 | 0.52 | 0.55 | 2.97** | 0.53 | 0.62 | 3.09** | 4.44** | 1.89** |
| ALP (µkat/L) | 0.41 | 0.44 | 0.73** | 3.35** | 0.40 | 0.72** | 3.55** | 3.91** | 1.80** | 0.39 | 0.45 | 0.55** | 2.93** | 0.41 | 0.59** | 2.96** | 4.06** | 1.20** |
| NAG (nkat/L) | 41 | 41 | 37 | 110** | 38 | 35 | 78** | 147** | 73** | 87 | 90 | 83 | 127** | 83 | 100 | 126** | 152** | 96 |
| GGT (nkat/L) | 35 | 38 | 51** | 76** | 34 | 48** | 66** | 83** | 78** | 41 | 42 | 50* | 117** | 46 | 63** | 106** | 125** | 105** |

Statistical analysis: Wilcoxon test (one-sided) *: p < 0.05; **: p < 0.01, values are given as mean

Table S12: Adverse hematological parameters of adult animals after 90-day inhalation exposure

|  | **Male animals (n=10)** | | | | | | | | | **Female animals (n=10)** | | | | | | | | |
| --- | --- | --- | --- | --- | --- | --- | --- | --- | --- | --- | --- | --- | --- | --- | --- | --- | --- | --- |
|  | **control** | **uZnO** | | | **cZnO** | | | **µZnO** | **ZnSO_4_** | **control** | **uZnO** | | | **cZnO** | | | **µZnO** | **ZnSO_4_** |
| Concentration (mg/m³) | **0** | **0.5** | **2** | **10** | **0.5** | **2** | **10** | **10** | **22** | **0** | **0.5** | **2** | **10** | **0.5** | **2** | **10** | **10** | **22** |
| White blood cells (giga/L) | 4.13 | 4.25 | 4.57 | 5.13 | 4.41 | 4.36 | 5.86** | 4.71 | 4.95 | 3.26 | 3.20 | 3.35 | 3.42 | 3.07 | 3.53 | 3.40 | 3.27 | 3.18 |
| Absolute neutrophils (giga/L) | 1.18 | 1.40 | 1.27 | 1.62 | 1.33 | 1.18 | 1.65** | 1.33 | 1.35 | 0.74 | 0.65 | 0.74 | 0.95* | 0.66 | 0.99 | 0.90 | 0.97 | 0.68 |
| Absolute lymphocytes (giga/L) | 2.65 | 2.55 | 3.00 | 3.21 | 2.83 | 2.91 | 3.96** | 3.12 | 3.28 | 2.26 | 2.30 | 2.38 | 2.19 | 2.18 | 2.27 | 2.21 | 2.10 | 2.25 |

Statistical analysis: Wilcoxon test (one-sided) *: p < 0.05; **: p < 0.01, values are given as mean

Table S13: Incidence and severity of main histological findings in lungs of adult animals after 90-day inhalation exposure to uZnO, cZnO, µZnO, and ZnSO_4_

|  | **Male animals (n=10)** | | | | | | | | | **Female animals (n=10)** | | | | | | | | |
| --- | --- | --- | --- | --- | --- | --- | --- | --- | --- | --- | --- | --- | --- | --- | --- | --- | --- | --- |
|  | **control** | **uZnO** | | | **cZnO** | | | **µZnO** | **ZnSO_4_** | **control** | **uZnO** | | | **cZnO** | | | **µZnO** | **ZnSO_4_** |
| Concentration (mg/m³) | **0** | **0.5** | **2** | **10** | **0.5** | **2** | **10** | **10** | **22** | **0** | **0.5** | **2** | **10** | **0.5** | **2** | **10** | **10** | **22** |
| No. of animals examined | 10 | 10 | 10 | 10 | 10 | 10 | 10 | 10 | 10 | 10 | 10 | 10 | 10 | 10 | 10 | 10 | 10 | 10 |
| **Lungs** | | | | | | | | | | | | | | | | | | |
| Foamy macrophages, alveolar | 0 | 0 | 5* | 10** | 0 | 5* | 10** | 10** | 10** | 0 | 0 | 0 | 10** | 0 | 0 | 10** | 10** | 10** |
| - Grade 1 | 0 | 0 | 5 | 0 | 0 | 5 |  | 1 | 0 | 0 | 0 | 0 | 0 | 0 | 0 | 1 | 2 | 6 |
| - Grade 2 | 0 | 0 | 0 | 4 | 0 | 0 | 4 | 6 | 5 | 0 | 0 | 0 | 2 | 0 | 0 | 5 | 5 | 4 |
| - Grade 3 | 0 | 0 | 0 | 5 | 0 | 0 | 5 | 2 | 5 | 0 | 0 | 0 | 8 | 0 | 0 | 4 | 3 | 0 |
| - Grade 4 | 0 | 0 | 0 | 1 | 0 | 0 | 1 | 1 | 0 | 0 | 0 | 0 | 0 | 0 | 0 | 0 | 0 | 0 |
| Debris, cellular, (multi)focal | 0 | 0 | 0 | 10** | 0 | 0 | 10** | 10** | 10** | 0 | 0 | 0 | 10** | 0 | 0 | 10** | 10** | 10** |
| - Grade 1 | 0 | 0 | 0 | 0 | 0 | 0 | 0 | 1 | 6 | 0 | 0 | 0 | 3 | 0 | 0 | 5 | 3 | 9 |
| - Grade 2 | 0 | 0 | 0 | 3 | 0 | 0 | 3 | 3 | 3 | 0 | 0 | 0 | 5 | 0 | 0 | 4 | 2 | 1 |
| - Grade 3 | 0 | 0 | 0 | 6 | 0 | 0 | 6 | 4 | 1 | 0 | 0 | 0 | 2 | 0 | 0 | 1 | 5 | 0 |
| - Grade 4 | 0 | 0 | 0 | 1 | 0 | 0 | 1 | 2 | 0 | 0 | 0 | 0 | 0 | 0 | 0 | 0 | 0 | 0 |
| Infiltration, neutrophils, (multi)focal | 0 | 0 | 1 | 10** | 0 | 0 | 10** | 10** | 10** | 0 | 0 | 0 | 10** | 0 | 0 | 10** | 10** | 10** |
| - Grade 1 | 0 | 0 | 1 | 1 | 0 | 0 | 1 | 5 | 9 | 0 | 0 | 0 | 1 | 0 | 0 | 5 | 5 | 9 |
| - Grade 2 | 0 | 0 | 0 | 7 | 0 | 0 | 9 | 3 | 1 | 0 | 0 | 0 | 9 | 0 | 0 | 5 | 5 | 1 |
| - Grade 3 | 0 | 0 | 0 | 2 | 0 | 0 | 0 | 2 | 0 | 0 | 0 | 0 | 0 | 0 | 0 | 9 | 0 | 0 |
| Hyperplasia, type II pneumocytes, (multi)focal | 0 | 0 | 0 | 9** | 0 | 0 | 8** | 6** | 7** | 0 | 0 | 0 | 10** | 0 | 0 | 8** | 8** | 9** |
| - Grade 1 | 0 | 0 | 0 | 5 | 0 | 0 | 8 | 3 | 6 | 0 | 0 | 0 | 5 | 0 | 0 | 1 | 5 | 7 |
| - Grade 2 | 0 | 0 | 0 | 4 | 0 | 0 | 0 | 3 | 1 | 0 | 0 | 0 | 5 | 0 | 0 | 0 | 3 | 2 |

Statistical analysis: Fisher’s exact test (one-sided+), *: p < 0.05; **: p < 0.01

Table S14: Incidence and severity of main histological findings in larynx and nasal cavity of adult animals after 90-day inhalation exposure to uZnO, cZnO, µZnO, and ZnSO_4_

|  | **Male animals (n=10)** | | | | | | | | | **Female animals (n=10)** | | | | | | | | |
| --- | --- | --- | --- | --- | --- | --- | --- | --- | --- | --- | --- | --- | --- | --- | --- | --- | --- | --- |
|  | **control** | **uZnO** | | | **cZnO** | | | **µZnO** | **ZnSO_4_** | **control** | **uZnO** | | | **cZnO** | | | **µZnO** | **ZnSO_4_** |
| Concentration (mg/m³) | **0** | **0.5** | **2** | **10** | **0.5** | **2** | **10** | **10** | **22** | **0** | **0.5** | **2** | **10** | **0.5** | **2** | **10** | **10** | **22** |
| No. of animals examined | 10 | 10 | 10 | 10 | 10 | 10 | 10 | 10 | 10 | 10 | 10 | 10 | 10 | 10 | 10 | 10 | 10 | 10 |
| **Larynx** | | | | | | | | | | | | | | | | | | |
| Epithelial alteration, (multi)focal | 1 | 1 | 0 | 6* | 0 | 2 | 2 | 1 | 0 | 0 | 0 | 1 | 4* | 0 | 1 | 3 | 0 | 0 |
| - Grade 1 | 1 | 1 | 0 | 6 | 0 | 2 | 2 | 1 | 0 | 0 | 0 | 1 | 4 | 0 | 1 | 3 | 0 | 0 |
| Metaplasia, squamous, (multi)focal | 0 | 0 | 0 | 0 | 0 | 0 | 0 | 0 | 10** | 0 | 0 | 0 | 0 | 0 | 0 | 0 | 0 | 10** |
| - Grade 1 | 0 | 0 | 0 | 0 | 0 | 0 | 0 | 0 | 3 | 0 | 0 | 0 | 0 | 0 | 0 | 0 | 0 | 1 |
| - Grade 2 | 0 | 0 | 0 | 0 | 0 | 0 | 0 | 0 | 7 | 0 | 0 | 0 | 0 | 0 | 0 | 0 | 0 | 9 |
| Inflammatory cell infiltrates (multi)focal | 0 | 0 | 0 | 0 | 0 | 0 | 0 | 0 | 1 | 0 | 0 | 0 | 0 | 0 | 0 | 0 | 0 | 9** |
| - Grade 1 | 0 | 0 | 0 | 0 | 0 | 0 | 0 | 0 | 1 | 0 | 0 | 0 | 0 | 0 | 0 | 0 | 0 | 5 |
| - Grade 2 | 0 | 0 | 0 | 0 | 0 | 0 | 0 | 0 | 0 | 0 | 0 | 0 | 0 | 0 | 0 | 0 | 0 | 4 |
| Erosion/ulcer | 0 | 0 | 0 | 0 | 0 | 0 | 0 | 0 | 0 | 0 | 0 | 0 | 0 | 0 | 0 | 0 | 0 | 1 |
| **Nasal cavity** | | | | | | | | | | | | | | | | | | |
| Degen./regen. olfactory epithelium (level III) | 0 | 0 | 1 | 5* | 0 | 3 | 3 | 0 | 10** | 0 | 1 | 0 | 8** | 0 | 1 | 5* | 0 | 10** |
| - Grade 1 |  |  | 1 | 5 | 0 | 2 | 3 | 0 | 5 | 0 |  | 0 | 7 | 0 | 0 | 5 | 0 | 5 |
| - Grade 2 |  |  |  |  | 0 | 1 |  | 0 | 5 | 0 | 1 | 0 | 1 | 0 | 1 | 0 | 0 | 5 |
| Degen./regen. olfactory epithelium (level IV) | 0 | 0 | 1 | 9** | 0 | 1 | 6** | 0 | 10** | 0 | 1 | 0 | 10** | 0 | 1 | 10** | 0 | 10** |
| - Grade 1 |  |  | 1 | 6 | 0 |  | 6 | 0 | 1 | 0 | 0 | 0 | 10 | 0 | 1 | 9 | 0 | 1 |
| - Grade 2 |  |  |  | 3 | 0 | 1 |  | 0 | 8 | 0 | 0 | 0 | 0 | 0 |  | 1 | 0 | 8 |
| - Grade 3 |  |  |  |  | 0 |  |  | 0 | 1 | 0 | 0 | 0 | 0 | 0 | 0 | 0 | 0 | 1 |

Statistical analysis: Fisher’s exact test (one-sided+), *: p < 0.05; **: p < 0.01

Table S15: Reproductive performance of adults and litters during inhalation exposure to uZnO, cZnO, µZnO, and ZnSO_4_

|  | control | uZnO | | | cZnO | | | µZnO | ZnSO_4_ |
| --- | --- | --- | --- | --- | --- | --- | --- | --- | --- |
| Concentration (mg/m³) | **0** | **0.5** | **2** | **10** | **0.5** | **2** | **10** | **10** | **22** |
| Estrous cycle length (d) |  | 3.9 | 3.9 | 4.0 | 4.0 | 3.9 | 3.9 | 4.0 | 4.0 |
| Male mating index (%) | 100 | 100 | 100 | 100 | 100 | 100 | 100 | 100 | 100 |
| Male fertility index (%) | 100 | 100 | 100 | 100 | 100 | 100 | 87.5 | 100 | 100 |
| Female mating index (%) | 100 | 100 | 100 | 100 | 100 | 100 | 100 | 100 | 100 |
| Female fertility index (%) | 100 | 100 | 100 | 100 | 100 | 100 | 87.5 | 100 | 100 |
| Gestation index (%) | 100 | 100 | 100 | 100 | 100 | 100 | 100 | 100 | 100 |
| Gestation duration (d) | 22.2 | 22.1 | 22.2 | 22.1 | 22.4 | 22.2 | 21.9 | 22.2 | 22.1 |
| Implantation sites | 13.2 | 12.0 | 12.8 | 12.2 | 12.4 | 12.3 | 12.5 | 12.5 | 12.1 |
| Post implantation loss (%) | 2.8 | 3.6 | 6.5 | 11.5* | 5.7 | 5.4 | 5.6 | 7.7* | 6.5 |
| Liveborn pups (%) | 100 | 100 | 98.3 | 98.6 | 98.1 | 97.9 | 100 | 100 | 99.6 |

Data are shown as mean if not indicated otherwise, *: p < 0.05; **: p < 0.01
Kruskal-Wallis (two-sided) and Wilcoxon test (two-sided): estrous cycle length
Wilcoxon test with Bonferroni-Holm adjustment: implantation sites, liveborn pups ((one-sided-) and post implantation loss (one-sided+)
Fisher’s Exact test (one-sided): mating, fertility, gestation indices
Dunnett test (two-sided): gestation duration

Table S16: Developmental toxicity of F1 progeny after exposure to uZnO, cZnO, µZnO, and ZnSO_4_

|  | control | uZnO | | | cZnO | | | µZnO | ZnSO_4_ |
| --- | --- | --- | --- | --- | --- | --- | --- | --- | --- |
| Concentration (mg/m³) | **0** | **0.5** | **2** | **10** | **0.5** | **2** | **10** | **10** | **22** |
| Viability index (%) | 99.5 | 99.0 | 100 | 100 | 95.7 | 97.9 | 100 | 99.6 | 99.5 |
| Lactation index (%) | 100 | 100 | 100 | 100 | 100 | 98.8 | 100 | 100 | 100 |

Data are shown as mean if not indicated otherwise. Statistical analysis: Wilcoxon test (one-sided-) with Bonferroni-Holm adjustment.

Table S17: Histological findings in the respiratory tract of pups (PND 22) in the modified 90-day repeated dose inhalation study

|  | **Male pups (n=5)** | | | | | | | | | **Female pups (n=5)** | | | | | | | | |
| --- | --- | --- | --- | --- | --- | --- | --- | --- | --- | --- | --- | --- | --- | --- | --- | --- | --- | --- |
|  | **control** | **uZnO** | | | **cZnO** | | | **µZnO** | **ZnSO_4_** | **control** | **uZnO** | | | **cZnO** | | | **µZnO** | **ZnSO_4_** |
| **Concentration (mg/m³)** | **0** | **0.5** | **2** | **10** | **0.5** | **2** | **10** | **10** | **22** | **0** | **0.5** | **2** | **10** | **0.5** | **2** | **10** | **10** | **22** |
| No. of animals examined | 5 | 5 | 5 | 5 | 5 | 5 | 5 | 5 | 5 | 5 | 5 | 5 | 5 | 5 | 5 | 5 | 5 | 5 |
| **Lungs** | | | | | | | | | | | | | | | | | | |
| Debris, cellular, (multi)focal | 0 | 0 | 0 | 0.4 | 0 | 0 | 0.2 | 1** | 0.4 | 0 | 0 | 0 | 0.8* | 0 | 0 | 0.8* | 1** | 0.2 |
| Infiltration, neutrophils, (multi)focal | 0 | 0 | 0 | 0.2 | 0 | 0 | 0.2 | 0.4 | 0.4 | 0 | 0 | 0 | 0.4 | 0 | 0 | 0.2 | 0.6 | 0.2 |
| **Nasal cavity** | | | | | | | | | | | | | | | | | | |
| Degen./regen. olfactory epithelium | 0 | 0 | 0 | 0 | 0.2 | 0 | 1.4* | 0.2 | 0.2 | 0 | 0 | 0 | 0 | 0 | 0.4 | 0.8 | 0.2 | 0.2 |

Statistical analysis: Fisher’s exact test (one-sided+) was performed based on incidences of the findings. *: p < 0.05; **: p < 0.01

Table S18: Incidence and severity of main histological findings in the respiratory tract of pups (PND 22) in the modified 90-day repeated dose inhalation study

|  | **Male pups (n=5)** | | | | | | | | | **Female pups (n=5)** | | | | | | | | |
| --- | --- | --- | --- | --- | --- | --- | --- | --- | --- | --- | --- | --- | --- | --- | --- | --- | --- | --- |
|  | **control** | **uZnO** | | | **cZnO** | | | **µZnO** | **ZnSO_4_** | **control** | **uZnO** | | | **cZnO** | | | **µZnO** | **ZnSO_4_** |
| **Concentration (mg/m³)** | **0** | **0.5** | **2** | **10** | **0.5** | **2** | **10** | **10** | **22** | **0** | **0.5** | **2** | **10** | **0.5** | **2** | **10** | **10** | **22** |
| No. of animals examined | 5 | 5 | 5 | 5 | 5 | 5 | 5 | 5 | 5 | 5 | 5 | 5 | 5 | 5 | 5 | 5 | 5 | 5 |
| **Lungs** | | | | | | | | | | | | | | | | | | |
| Debris, cellular, (multi)focal | 0 | 0 | 0 | 2 | 0 | 0 | 1 | 5** | 2 | 0 | 0 | 0 | 4* | 0 | 0 | 4* | 5** | 1 |
| - Grade 1 |  |  |  | 2 |  |  | 1 | 5 | 2 |  |  |  | 4 |  |  | 4 | 5 | 1 |
| Infiltration, neutrophils, (multi)focal | 0 | 0 | 0 | 1 | 0 | 0 | 1 | 2 | 2 | 0 | 0 | 0 | 2 | 0 | 0 | 1 | 3 | 1 |
| - Grade 1 |  |  |  | 1 |  |  | 1 | 2 | 2 |  |  |  | 2 |  |  | 1 | 3 | 1 |
| **Nasal cavity** | | | | | | | | | | | | | | | | | | |
| Degen./regen. olfactory epithelium | 0 | 0 | 0 | 0 | 1 | 0 | 4* | 1 | 1 | 0 | 0 | 0 | 0 | 0 | 2 | 2 | 1 | 1 |
| - Grade 1 |  |  |  |  | 1 |  | 2 | 1 | 1 |  |  |  |  |  | 2 |  | 1 | 1 |
| - Grade 2 |  |  |  |  |  |  | 1 |  |  |  |  |  |  |  |  | 2 |  |  |
| - Grade 3 |  |  |  |  |  |  | 1 |  |  |  |  |  |  |  |  |  |  |  |

Statistical analysis: Fisher’s exact test (one-sided+), *: p < 0.05; **: p < 0.01

Table S19: Summary of ICP-OES analysis of zinc content in lungs, liver, heart, and brain of parental male and female Wistar rats after 90-day inhalation exposure to uZnO, cZnO, µZnO, and ZnSO_4_ (all values as µg)

|  |  | **Male (n=3)** | | | | | **Female (n=3)** | | | | |
| --- | --- | --- | --- | --- | --- | --- | --- | --- | --- | --- | --- |
|  |  | **control** | **uZnO** | **cZnO** | **µZnO** | **ZnSO_4_** | **control** | **uZnO** | **cZnO** | **µZnO** | **ZnSO_4_** |
| **Concentration**  **(mg/m³)** |  | **0** | **10** | **10** | **10** | **22** | **0** | **10** | **10** | **10** | **22** |
| Lung |  | 21 | 38 | 33 | 38 | 28 | 19 | 25 | 29 | 39 | 26 |
|  |  | 19 | 37 | 29 | 43 | 33 | 18 | 25 | 25 | 26 | 27 |
|  |  | 20 | 34 | 36 | 42 | 26 | 18 | 27 | 23 | 31 | 26 |
|  | mean | 20.0 | 36.3** | 32.7* | 41.0** | 29.0* | 18.3 | 25.7** | 25.7* | 32.0 | 26.3** |
|  | SD | 0.8 | 1.7 | 2.9 | 2.2 | 2.9 | 0.5 | 0.9 | 2.5 | 5.4 | 0.5 |
|  | Welch t test |  | 0.001 | 0.018 | 0.002 | 0.041 |  | 0.002 | 0.049 | 0.068 | 0.000 |
| Liver |  | 360 | 370 | 320 | 370 | 350 | 380 | 370 | 350 | 370 | 310 |
|  |  | 300 | 370 | 350 | 380 | 450 | 300 | 320 | 190 | 330 | 330 |
|  |  | 380 | 350 | 390 | 400 | 260 | 360 | 330 | 210 | 330 | 290 |
|  | mean | 346.7 | 363.3 | 353.3 | 383.3 | 353.3 | 346.7 | 340.0 | 250.0* | 343.3 | 310.0 |
|  | SD | 34.0 | 9.4 | 28.7 | 12.5 | 77.6 | 34.0 | 21.6 | 71.2 | 18.9 | 16.3 |
|  | Welch t test |  | 0.565 | 0.843 | 0.263 | 0.919 |  | 0.828 | 0.186 | 0.911 | 0.266 |
| Heart |  | 18 | 19 | 17 | 18 | 16 | 15 | 13 | 12 | 15 | 12 |
|  |  | 16 | 19 | 15 | 20 | 22 | 13 | 11 | 10 | 12 | 15 |
|  |  | 16 | 16 | 18 | 17 | 14 | 13 | 13 | 11 | 13 | 13 |
|  | mean | 16.7 | 18.0 | 16.7 | 18.3 | 17.3 | 13.7 | 12.3 | 11.0* | 13.3 | 13.3 |
|  | SD | 0.9 | 1.4 | 1.2 | 1.2 | 3.4 | 0.9 | 0.9 | 0.8 | 1.2 | 1.2 |
|  | Welch t test |  | 0.338 | 1.000 | 0.211 | 0.811 |  | 0.230 | 0.040 | 0.779 | 0.779 |
| Brain |  | 26 | 27 | 27 | 27 | 27 | 24 | 24 | 23 | 25 | 27 |
|  |  | 28 | 27 | 25 | 28 | 28 | 23 | 23 | 24 | 24 | 24 |
|  |  | 26 | 28 | 28 | 28 | 26 | 25 | 24 | 24 | 22 | 24 |
|  | mean | 26.7 | 27.3 | 26.7 | 27.7 | 27.0 | 24.0 | 23.7 | 23.7 | 23.7 | 25.0 |
|  | SD | 0.9 | 0.5 | 1.2 | 0.5 | 0.8 | 0.8 | 0.5 | 0.5 | 1.2 | 1.4 |
|  | Welch t test |  | 0.438 | 1.000 | 0.274 | 0.725 |  | 0.649 | 0.649 | 0.770 | 0.447 |
| Olfactory bulb |  | n.d. | n.d. | n.d. | n.d. | n.d. | n.d. | n.d. | n.d. | n.d. | n.d. |
|  |  | n.d. | n.d. | n.d. | n.d. | n.d. | n.d. | n.d. | n.d. | n.d. | n.d. |
|  |  | n.d. | 4 | n.d. | n.d. | n.d. | n.d. | n.d. | n.d. | n.d. | n.d. |

Statistical analysis: Welch t test, two sided *: p < 0.05; **: p < 0.01,
n.d. = not detectable (detection limit was 3 µg)
